# MosaicSolver: a tool for determining recombinants of viral genomes from pileup data

**DOI:** 10.1093/nar/gku524

**Published:** 2014-08-12

**Authors:** Graham R. Wood, Eugene V. Ryabov, Jessica M. Fannon, Jonathan D. Moore, David J. Evans, Nigel Burroughs

**Affiliations:** 1 Warwick Systems Biology Centre, Senate House, University of Warwick, Coventry, CV4 7AL, UK; 2 School of Life Sciences, University of Warwick, Coventry, CV4 7AL, UK

## Abstract

Viral recombination is a key evolutionary mechanism, aiding escape from host immunity, contributing to changes in tropism and possibly assisting transmission across species barriers. The ability to determine whether recombination has occurred and to locate associated specific recombination junctions is thus of major importance in understanding emerging diseases and pathogenesis. This paper describes a method for determining recombinant mosaics (and their proportions) originating from two parent genomes, using high-throughput sequence data. The method involves setting the problem geometrically and the use of appropriately constrained quadratic programming. Recombinants of the honeybee deformed wing virus and the *Varroa destructor* virus-1 are inferred to illustrate the method from both siRNAs and reads sampling the viral genome population (cDNA library); our results are confirmed experimentally. Matlab software (MosaicSolver) is available.

## INTRODUCTION

Recombination provides a mechanism for the rapid evolution of viruses, being implicated in the emergence of many recent pathogenic viral strains in public health and agriculture. Recent outbreaks of avian influenza (1,2) have implicated a recombinant event as a primary cause, honeybee population decline is associated with a deformed wing virus (DWV) recombinant (3,4) and current global potato crop devastation is caused by the highly pathogenic Y NTN virus strain (5,6). Further, human immunodeficiency virus continues to evolve with recombinants now predominating in many geographical areas exacerbating control measures (7), whilst recombination has also become a focus as a potential risk factor in the use of live attenuated virus vaccines (8). These are all examples of virulence shifts, the recombined virus acquiring new capabilities such as escape from the immune system, drug resistance, increased transmission rates, changes in tissue tropism or acquisition of novel host tropism allowing cross-species transmission. Despite these evolutionary advantages, a recent review (9) suggests that recombination of ribonucleic acid (RNA) viruses may not be a selected trait but a biproduct of the RNA polymerase mechanism.

Recombination is mediated through co-infection of a cell and can in principle occur anywhere along the genome, although recombination points do have preferred hotspots (10–12). For instance, recombination in poliovirus was shown to be associated with RNA structure and exhibits a GC content bias over an infection cycle (11), whilst protein incompatibility and selection pressure on regulatory, maturation or associated protein functions are likely to add a further layer of selection for the location of recombination points, producing the well-known bias between structural and nonstructural genes (10). Furthermore, recent evidence indicates that the recombination mechanism is biphasic, involving distinct crossover and resolution events (12). Mapping these locations is vital for identifying the determinants of recombination and understanding the characteristics of emergent strains. Identification of recombinants within a population of mixed viral genomes, together with their abundance, is thus a problem of fundamental significance.

Detection of recombinants, especially when there is no prior knowledge of recombination junctions (which would allow construction of suitable primers), is difficult, particularly if more than one recombinant progeny form is present. Next-generation sequencing (NGS) approaches provide a new opportunity to perform this task; new challenges arise however, particularly in the reconstruction of the underlying genomes from small sequences [typically less than 100 nucleotides (nt)]. In this paper, we present a novel approach to identify, characterize, quantify and assess the statistical significance of recombinant genomes in NGS sampling of population mixtures. Throughout we assume that the parent viral genomes can be globally aligned and that any recombination involves exchange of homologous regions.

The current work was motivated by ongoing investigations into honeybees (*Apis mellifera*) infested with a parasitic mite (*Varroa destructor*). The latter acts as a vector for a range of pathogenic viruses (13–15), the most important of which (both in terms of the individual honeybee and the penetration of colonies in the UK) are viruses related to the deformed wing virus (DWV-like viruses), which include DWV itself and its relative *Varroa destructor* virus-1 (VDV-1) that share an 84% nt (95% amino acid) identity. The latter was first extracted from *Varroa* mites (16). High levels of DWV-like viruses are associated primarily with deformed wings, including atrophied wing development and abdominal stunting (17). DWV-like viruses are endemic in honeybees worldwide, usually being asymptomatic, with the virus presumably being controlled and thus not reaching harmful levels; however, it has been reported to be responsible for overwintering colony demise, although the cause of the shift from a benign to a pathogenic infection is unknown. Co-infection of either the host honeybee or the mite with DWV and VDV-1 may result in the formation of recombinants between the two viruses. Such recombinants could accumulate to high levels and it is hypothesized that one or a very limited range of such recombinant forms is responsible for colony demise (3–4,18). Thus, ascertaining the recombinant profile within a population is a problem of key significance to food security. Different recombinants of DWV and VDV-1 strains have been reported (3,19). This makes the identification of DWV/VDV-1 recombinants a good system for the development of methods for recombinant identification, especially as mixed infections (parental and recombinant genomes) are present in the same individual.

As part of the analysis of the virological consequences of infesting *Varroa*-free colonies with mites we acquired two types of high-throughput sequence data, specifically sequencing of small interfering RNAs (siRNA; single-stranded RNAs that were generated as a result of the action of several components of the honeybee RNAi pathway) and short reads from the viral genome population [amplified complementary deoxyribonucleic acid (cDNA)], both extracted from *Varroa*-exposed, high viral load pupae. These independently generated data sets allowed us to investigate the development of a method to disentangle recombinant populations using both short, 21–22-nt reads (siRNA) and long, around 100-nt (cDNA) reads. These data, arising from parent genomes and potential recombinants within the viral population, allow the relative abundance of DWV and VDV-1 reads to be determined in any continuous segment of the genome, hereafter termed the segment proportions. All alignments used to produce segment proportions are exact, with no indels, and aligned to only one of the parent genomes (DWV and VDV-1 in our case). Use of such uniquely aligned reads is necessary, since reads capable of alignment to both parent genomes are allocated randomly to either genome in software such as Bowtie (20), masking the true ratio. We assume that these exact alignments give an accurate estimate of the local genome proportions.

**Figure 1.**
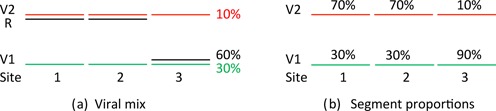
The unravelling problem. (**a**) A 30%, 10% and 60% mix of parent genomes V1, V2 and recombinant genome R which is V2 in the first two segments and V1 in the third segment. (**b**) The two parent genomes, with genomes partitioned into three segments, have segment proportions (V2/(V1 + V2)) of (0.7, 0.7, 0.1). Given the information in (b) the challenge is to find the recombinant genome R and the genome proportions in (a).

From these segment proportions, we propose to estimate both the main recombinant genomes within the population (assumed to comprise a low number of recombinants) and their relative occurrence. We use a simple example to illustrate the geometrical setting for formulating the problem and the associated computations; this example will be revisited and extended later as the methodology is described. Consider a viral mix comprising quantities of two parent genomes V1 and V2 and a recombinant R occurring with proportions 30%, 10% and 60%, respectively, in the population. The two parent genomes are assumed aligned and the genome divided into three ‘segments’, consecutive sections of the common alignment covering the full genome. Suppose that R is V2 on the first two segments and V1 on the third, shown in Figure 1a. Across segments, the proportion of V2-aligned reads in the mix is (*p*_1_, *p*_2_, *p*_3_) = (0.7, 0.7, 0.1) as shown in Figure 1b. We are interested in the reverse problem, namely given the segment proportions of V2 along the genome, determine both the recombinant genome and the proportions of genomes V1, V2 and R in the virus population.

There appears to be no method to date in the literature addressing this recombinant identification problem. Methods addressing related challenges, however, do exist. There is an extensive literature describing statistical tests for detecting mosaic structure when parent genomes and putative mosaics are available, reviewed in (21). In a different vein, a series of three papers (22–24) develop methods for estimating the relative abundance and size of genomes in a metagenome. GAAS (Genome relative Abundance and Average Size), a Basic Local Alignment Search Tool-based approach, is introduced in (22) whilst Xia *et al.* in (23) build on GAAS to create model-based GRAMMy (Genome Relative Abundance using Mixture Model Theory). Finally, Lindner and Renard (24) present GASiC (Genome Abundance Similarity Correction) to improve estimation in the event of highly similar reference genomes; a matrix capturing alignment similarities between the reference genomes is used in a linear model to correct for genome similarity. In a separate but related research direction, Gong *et al.* (25) estimate the proportion of different cell types in a mixture of multiple cell types, using transcriptional profiling data. Closest in approach to the current work, however, is a method of Meinicke *et al.* (26); the weights of organisms in a microbial community are estimated by expressing the oligonucleotide probabilities of the metagenome as a convex combination over each component genome. In this, estimation of the weights is carried out using quadratic programming.

The layout of the paper is as follows. The methodology to unravel recombinant genome mixtures is described in the Materials and Methods section. In the Results section, we apply this algorithm to our honeybee data, both the siRNA data and viral sequence data, and examine accuracy on simulated data. A systematic approach to the search for the recombinant genomes is described and illustrated. Related questions of a blind search for recombination points, accuracy limits and identifiability of the set of recombinants are also discussed. Experimental validation of our predictions is presented in the Experimental validation of MosaicSolver predictions section. Discussion and Conclusion sections complete the paper.

## MATERIALS AND METHODS

### Sequence data

Three high-throughput honeybee data sets are used: a set of siRNA sequence data (ArrayExpress accession: E-MTAB-1671, as below), an RNA-seq data set sampling the viral genome from (3) and a mix of two recombinant genomes originally discussed in (3) and expanded on here.

#### siRNA data set

Small RNA was extracted from 14-day-old bee pupae taken from capped cells containing *Varroa* mites. For the siRNA analysis, reads were trimmed to remove adapter sequences and only reads 18 nt or longer were used. The reads were aligned to DWV and VDV-1 reference sequences (GenBank Accession numbers GU109335 and AY251269, respectively) using Bowtie (20). For alignment, we set a seed length of 16 and allowed no mismatches in the seed. Only sequences unambiguously aligned to a single reference sequence were used to produce pileup files, using SAMtools mpileup (27).

#### Recombinant genome mix data set

We produced a mix of the recombinant RNA genomes VDV-1_VVD_ and VDV-1_DVD_, described in (3), using *in vitro* RNA transcripts produced with the T7 mMESSAGEmMACHINE kit (Ambion) from plasmids containing full-length cDNAs of either VDV-1_VVD_ or VDV-1_DVD_. These were linearized using a unique restriction site downstream of the 3′ ends of the viral cDNA inserts. The RNA transcripts were purified using RNAeasy columns (Quiagen), quantified by spectrophotometry (Nanodrop), mixed 75% VDV-1_VVD_, 25% VDV-1_DVD_ and 8 466 404 reads sequenced using the Illumina platform protocol (‘Virus 59’, EBI Sequence Read Archive study accession PRJEB5249). Reads were aligned to the DWV and VDV-1 reference sequences using Bowtie 2 (28) using the ‘sensitive-local’ option with a seed length of 20 nt and allowing no mismatches. The pileup file was produced using SAMtools mpileup.

### Recombinant identification algorithm

For clarity, the algorithm is described in the context of parent genomes DWV and VDV-1; the generalization to any pair of similar genomes and possible recombinants is evident. The algorithm assumes that there is a global alignment of the parent genomes, permitting a small number of indels.

**Figure 2.**
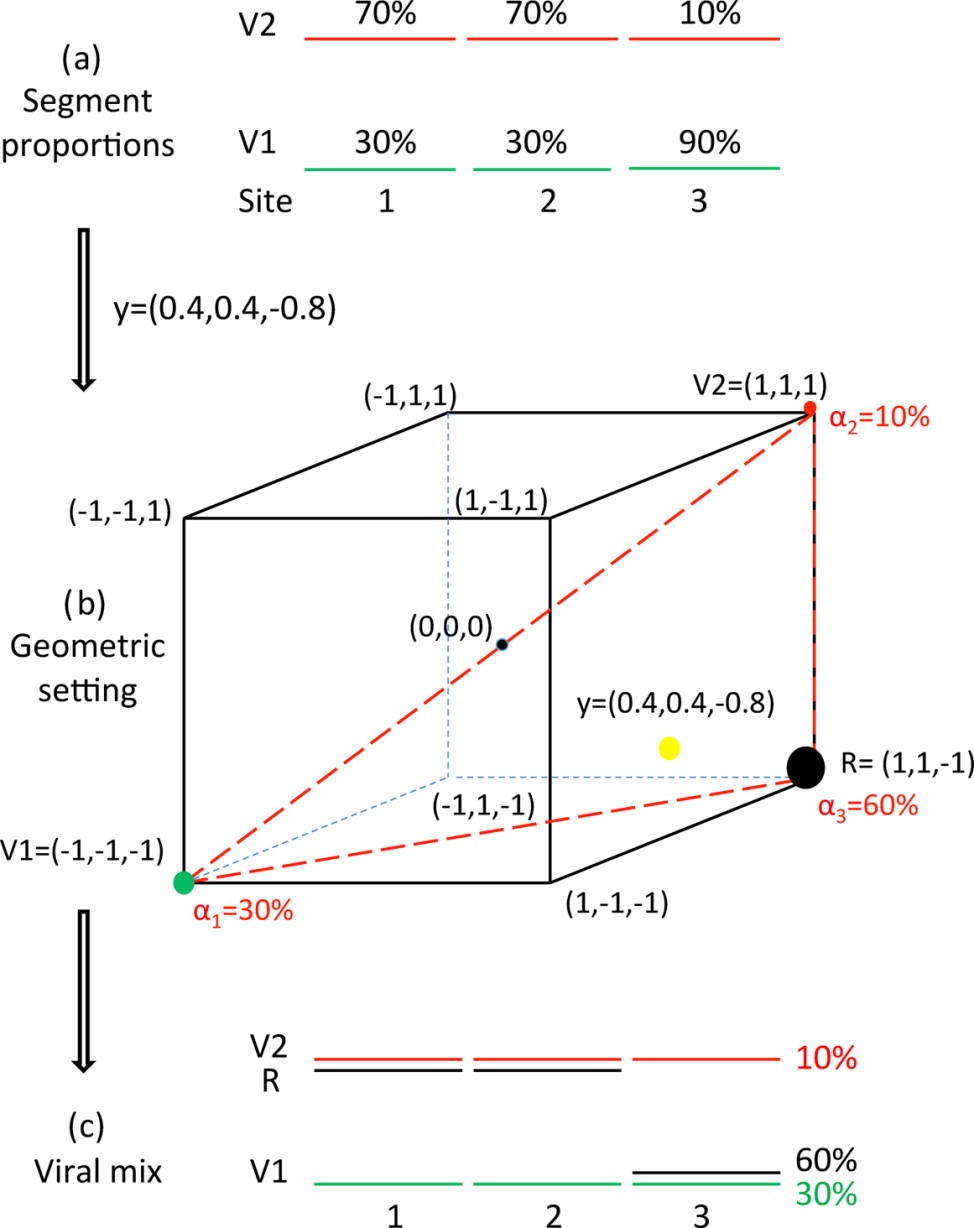
The passage from pileup proportion data to geometry to recombinants. (**a**) Genome segment proportions (*p*_1_, *p*_2_, *p*_3_) = (0.7, 0.7, 0.1). (**b**) The eight possible mosaics available for three segments are shown as the vertices of the cube in *R*^3^, with DWV = (−1, −1, −1) and VDV-1 = (1, 1, 1) at the extremes of a cube diameter. Genome proportions map to *y* = (*y*_1_, *y*_2_, *y*_3_) = (0.4, 0.4, −0.8) in the cube, which in this example lies in the triangle spanned by DWV, VDV-1 and the recombinant *R* = (1, 1, −1). The barycentric coordinates of *y* are α = (α_1_, α_2_, α_3_) = (0.3, 0.1, 0.6), their relative sizes illustrated as solid circles on the three vertices. (**c**) Reconstruction of the segment proportions *p* in the viral mix from the three vertices DWV, VDV-1 and R and their weights α.

We begin by aligning the DWV and VDV-1 genomes and nominating *n* − 1 possible recombination points, termed breakpoints, in the common alignment (choice of these breakpoints is discussed later). These artificially break the common genome into *n* segments, each segment ending immediately before a breakpoint, illustrated for *n* = 3 in Figure 2a. A recombinant R of the two genomes (informally termed a mosaic) is a choice of DWV or VDV-1 in each segment. A mosaic genome can be represented as a binary sequence of length *n*, with each component either ‘−1’ or ‘1’, according to whether it is from DWV or VDV-1, respectively. Such sequences are precisely the vertices of the hypercube *C*^*n*^ = [−1, 1]^*n*^ in *R*^*n*^, with DWV being the vertex (−1, −1, …, −1) and VDV-1 the vertex (1, 1, …, 1), as illustrated for *n* = 3 in Figure 2b.

Available reads are aligned to each of the DWV and VDV-1 genomes and pileup counts of exact alignments to a unique parent genome recorded. These counts are transferred to the common global alignment, then the total pileup counts calculated in each segment for each genome and finally segment proportions calculated. The VDV-1 proportion of all segment pileup counts are denoted as *p* = (*p*_1_, *p*_2_, …, *p*_*n*_). The measured segment proportions determine a point *y* = (*y*_1_, *y*_2_, …, *y*_*n*_) within the hypercube *C*^*n*^ and vice versa, where *y*_*i*_ = −1 + 2*p*_*i*_ for *i* = 1, …, *n*. For example, if *p*_1_ = 0.5 then DWV and VDV-1 are equally likely in the first segment and *y*_1_ = 0.

#### Finding a single recombinant

A viral mix made up precisely of DWV (vertex V1), VDV-1 (vertex V2) and a recombinant R can be represented, through its transformed segment proportions, by a point *y* = (*y*_1_, *y*_2_, …, *y*_*n*_) in the triangle within *C*^*n*^ spanned by these three vertices of the hypercube. Vertex weights (α_1_, α_2_, α_3_), summing to one, placed on the vertices of this triangle and with centre of mass *y* = α_1_*V*1 + α_2_*V*2 + α_3_*R*, are the population proportions of genomes DWV, VDV-1 and R; these weights are known as the barycentric coordinates of *y*.

These ideas are illustrated in Figure 2 using the example with *n* = 3 introduced in Figure 1. The segment proportions of (0.7, 0.7, 0.1) in (a) are mapped to *y* = (0.4, 0.4, −0.8) in the three-dimensional cube *C*^3^ = [−1, 1]^3^ in (b). DWV corresponds to vertex (−1, −1, −1) and VDV-1 to vertex (1,1,1); the six other possible recombinants correspond to the other vertices of the cube. Point *y* (in yellow) lies in the triangle spanned by DWV, VDV-1 and (1, 1, −1), highlighted in red in the figure. The vertex weights (α_1_, α_2_, α_3_) = (0.3, 0.1, 0.6) with centre of mass *y*, shown with appropriately coloured and sized solid circles, provide the genome weights. The algebraic relationship, *y* = α_1_*V*1 + α_2_*V*2 + α_3_*R*, is realized numerically as

\begin{eqnarray*} &&(0.4,0.4,-0.8)= \\ &&0.3(-1,-1,-1)+0.1(1,1,1)+0.6(1,1,-1). \end{eqnarray*}

Figure 2c then shows R and the genome weights in a more familiar genome format.

In general, for a mosaic comprising *n* segments, the segment proportions define a point in *C*^*n*^ and the task is to determine the best R and the associated genome weights (α_1_, α_2_, α_3_) on DWV, VDV-1 and R. In the previous example the data *y* lay in a triangle spanned by three vertices. Generally this will not be the case and we need to find the viral mix that most closely gives rise to the observed segment proportions, that is, the point *x* = (*x*_1_, *x*_2_, …, *x*_*n*_) in a triangle (spanned by DWV, VDV-1 and a recombinant) closest to the data point *y*. Specifically, when the segment pileup totals differ, we need to solve the weighted squared distance minimization problem

\begin{equation*} \min _{R} \min _{x \in {\rm co}\lbrace{\rm DWV},{\rm VDV} {\scriptsize-}1,{\rm R}\rbrace}\ \ \sum _{i=1}^n w_i (x_i-y_i)^2, \end{equation*}

where *R* is a vertex of *C*^*n*^, *x* lies in the convex hull (co) of {DWV,VDV-1,R} and *w*_*i*_ is the proportion of the total pileup data in segment *i* (this weighting ensures that the terms in the summation correctly reflect the pileup count in each segment). The recombinant R is set in turn to each of the 2^*n*^ − 2 vertices of *C*^*n*^ other than DWV and VDV-1. For each choice of R, efficient constrained quadratic programming methods and software are available to find *x*. The barycentric coordinates of this nearest point *x* in the triangle spanned by DWV, VDV-1 and R provide the proportions with which genomes DWV, VDV-1 and R occur in the mixture; the barycentric coordinates of *x* are found as the solution of a system of three linear equations in three variables. The weighted Euclidean distance from *x* to *y*, $\sqrt{\sum _{i=1}^n w_i (x_i-y_i)^2}$, is then minimal for that *R*. The analysis is then repeated for all vertices R and the vertex generating a triangle whose closest point *x* to *y* is in fact closest to *y* is selected as the optimal recombinant; this recombinant and the associated genome proportions are the best explanation of the data assuming that only one recombinant is present. The remainder is due to measurement noise and also the presence of additional lower frequency recombinants.

**Figure 3.**
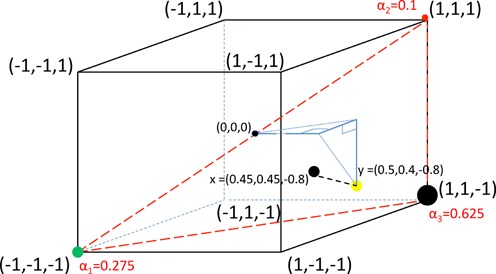
Single recombinant search with partially explained data. Here the data point *y* = (0.5, 0.4, −0.8) does not lie in a triangle spanned by DWV, VDV-1 and any other vertex. All vertices other than DWV and VDV-1 can be searched and the vertex, here (1, 1, −1), providing the minimum distance selected, it being the most likely single mosaic. The nearest point to *y* in this red triangle is *x* = (0.45, 0.45, −0.8) with barycentric coordinates α = (0.275, 0.1, 0.625). The minimum distance is 0.0707, small relative to the diameter of the cube, $2\sqrt{3}=3.4641$$2\sqrt{3}=3.4641$.

This general case is illustrated in Figure 3 where *y* = (0.5, 0.4, −0.8), moved so as to not lie in any triangle spanned by DWV, VDV-1 and a recombinant. The recombinant R whose associated triangle contains the point nearest to *y* is (1, 1, −1) with a closest point *x* = (0.45, 0.45, −0.8) at Euclidean distance 0.0707 from *y*. Weights α = (α_1_, α_2_, α_3_) = (0.275, 0.1, 0.625) recover *x*, but with discrepancy in the first two segments, as follows:

\begin{eqnarray*} &&0.275(-1, -1, -1)+0.1(1, 1, 1)+0.625(1, 1, -1)= \\ &&(0.45, 0.45, -0.8). \end{eqnarray*}

**Figure 4.**
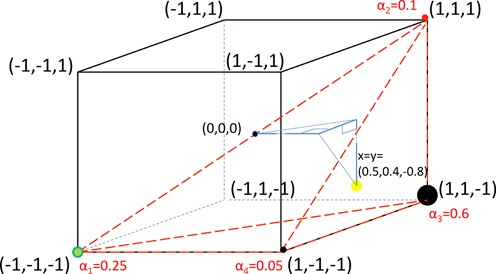
Two recombinant search with fully explained data. Data point *y* = (0.5, 0.4, −0.8) lies in the tetrahedron spanned by DWV, VDV-1, (1, 1 − 1) and (1, −1, −1). Associated vertex weights, displayed with solid circles, are (0.25, 0.1, 0.6, 0.05).

The discrepancy in this example can be explained by the existence of a second mosaic genome, Figure 4; the two mosaics (1, 1, −1) and (1, −1, −1) together with DWV and VDV-1 provide an exact fit for data point *y* = (0.5, 0.4, −0.8). The associated mosaic vertex weights are α = (0.25, 0.1, 0.6, 0.05).

#### Finding multiple recombinants

When seeking more than one recombinant, two search strategies are available. The computationally less expensive is a stepwise-forward serial (or sequential, one-at-a-time) approach, at each stage adding the mosaic most capable of explaining the observed segment proportions from amidst all possible remaining mosaics not in the span of those already chosen. Restricting the selection process to such mosaics ensures that the resulting mosaic weights are unique. The success of serial search rests on the assumption that mosaics already found in earlier recursions will remain in any solution involving a higher number of mosaics. This assumption may be incorrect and can be removed by using a computationally more expensive parallel (or simultaneous, all-at-once) fitting method. Such a parallel search for the most likely pair of mosaics involves searching all _*n* − 2_*C*_2_ tetrahedra (3-simplexes); a search of all _*n* − 2_*C*_*s* − 1_ *s*-simplexes finds the most likely *s* mosaics. The complexity of the serial method is linear in the number of recombinants to be found, whereas the parallel method is exponential in the number of recombinants to be found. A comparison of the accuracy of the two approaches is presented in the Identifiability section.

Matlab (version R2013a) software, called MosaicSolver, implementing both methods is available from the Warwick Systems Biology Centre software website at http://wsbc.warwick.ac.uk/software.

## RESULTS

We applied our geometric method, implemented in MosaicSolver, see the Materials and Methods section, to identify recombinants and their proportions in two experimental data sets. On siRNA data (21–22-nt reads), we illustrated the basic methodology for determining the composition of a viral population in terms of an unknown number of mosaic genomes, and also a refinement technique to locate the recombinant breakpoints. Applied to Illumina short read data from reverse-transcribed whole genome samples (∼100-nt reads) we compared our predictions against sequenced cloned genomes. To validate the method, we firstly analysed the robustness and accuracy of the method on simulated data, showing that accuracy is highest for a small number of recombinants; this is related to an identifiability problem. Secondly, we experimentally verified the approach in a variety of ways, which included the creation of a known mixture of two DWV/VDV-1 recombinant genomes followed by prediction of both the recombinants and their proportions from the NGS reads.

### Examples on pileup data

**Figure 5.**
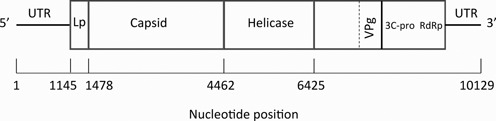
Map of the DWV genome [adapted from (29)] with nucleotide positions of individual features used to define recombinants indicated. Shown also are the untranslated regions (UTRs) and the location of regions coding for the viral proteins.

*Example 1: Viral recombinant generation using the siRNA data set.* An experiment was carried out to study the effect of DWV and the mite *Varroa* on siRNA composition (small interfering RNA, generated by the innate RNAi immune system), using high-throughput sequencing of siRNA in developing worker honeybees. Newly hatched bee larvae (day 3 after egg laying) were transferred from a *Varroa*-free colony with low DWV levels to a *Varroa*-infested colony with high levels of DWV in both bees and *Varroa* mites. All transferred larvae were exposed to the DWV strains present in the *Varroa*-infested colony via the food delivered by the nurse bees until their capping (day 9). Approximately half of these larvae were capped with *Varroa* mites that fed on haemolymph during pupal development, until sampling at the purple-eye stage (14 days after laying). The majority of mite-exposed pupae exhibited strikingly elevated DWV levels, at least 1000 times higher than seen in *Varroa*-free pupae (3). The siRNA reads sampled from eight of these honeybees, each showing high viral load, and that aligned exactly to just one of the DWV or VDV-1 genomes, were used to generate pileup counts at each nucleotide, from position 1 to position 10 129. A map of the DWV genome is shown in Figure 5.

#### Estimation of recombinants using coarse segmentation

**Figure 6.**
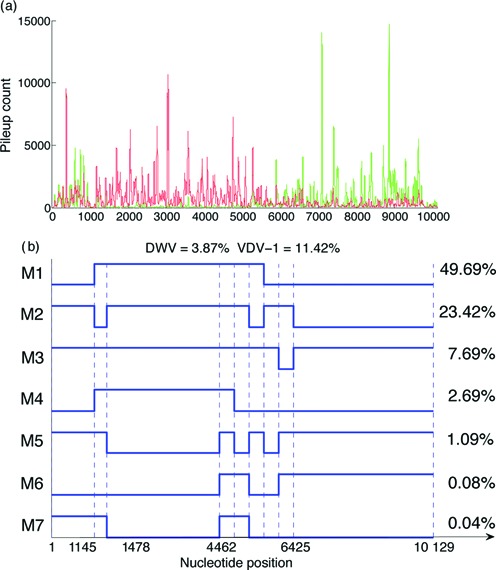
The siRNA pileup data and the first seven recombinant mosaics (M1–M7) found in the viral mix obtained from bees with high viral levels and exposed to the virus population through both food and mites. (**a**) siRNA pileup data, with DWV read pileup counts (based on 258 928 reads) shown in green and VDV-1 counts in red (based on 316 237 reads). (**b**) Recombinant mosaics. Five equal length segments were used in the helicase region together with breakpoints after the 5′ UTR and Lp regions, giving *n* = 9 segments; the serial search algorithm was used. Each mosaic is shown as a recombination of DWV (lower level) and VDV-1 (higher level) along the genome. Percentages of the parental genomes DWV and VDV-1 detected in the population are given at the top and mosaic percentages on the right.

Recombination in the capsid region is considered to be unlikely, so breakpoints were initially assigned at nucleotide positions 1145, 1478, 4462, 4855, 5247, 5640, 6032 and 6425. These correspond to the start of known functional domains in the genome (17,29), with 1145 being the start of the DWV open reading frame [the end of the 5′ untranslated region (UTR) and start of the leader protein (Lp) coding region], 1478 marking the end of the Lp coding region and start of the structural component and 4462 marking the end of the capsid (structural proteins) coding region and start of the helicase-coding region. Previous studies have indicated that a significant number of recombination events occur within the helicase (3,30); hence, we added additional breakpoints by subdividing the helicase-coding region into five segments (breakpoints at nucleotides 4462–6425 inclusive). Thus, *n* = 9 giving the hypercube *C*^9^ with 2^9^ = 512 vertices, i.e. there are 510 possible mosaic genomes. The siRNA pileup data and the first seven recombinants found using a serial search, together with their proportions, are presented in Figure 6.

**Figure 7.**
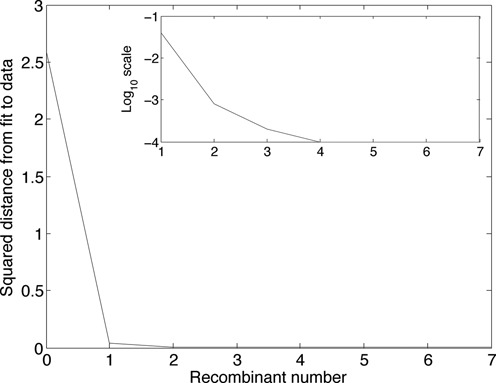
The squared distance between data point *y* and fitted point *x*, plotted against the number of recombinants fitted. In the example, the drops are 2.545, 3.971 × 10^−2^, 6.023 × 10^−4^, 1.267 × 10^−4^, 3.783 × 10^−5^, 3.451 × 10^−7^ and 2.143 × 10^−8^, the corresponding remaining squared distances are 4.053 × 10^−2^, 8.171 × 10^−4^, 2.148 × 10^−4^, 8.820 × 10^−5^, 5.036 × 10^−5^, 5.002 × 10^−5^ and 5.000 × 10^−5^, whence the successive *F*-values are 62.79, 48.60, 2.804, 1.437, 0.7512, 0.0069 and 0.0004, making the first two recombinants significant at the 1% level. The inset plots log_10_ of the squared distance against the number of recombinants.

#### Significance testing of recombinants

The squared distance between the data point *y* and the best fitting point in *C*^9^ for each successively fitted model is plotted against the number of recombinants in the model in Figure 7. A substantial fall indicates that the added recombinant explains a substantial proportion of the variation in the segment proportions; hence, this suggests that the first two recombinants shown in Figure 6 are real, whilst the rest are probably insignificant. A statistical test can be constructed by noting that each drop in the graph is a sum of independent squared differences (one for each segment). We consider the ratio of the drop to the remaining squared distance following a similar strategy used in linear regression (31, Ch. 14]; a ratio of distances is invariant under scale changes of the cube, necessary since the absolute distance has no meaning. If both numerator and denominator are noise (assumed Gaussian), the ratio will follow a Fisher–Snedecor *F*_*n*, *n*_ distribution. On the other hand, if the first has a substantial signal, the ratio will be inflated; given that the second may also include a signal component, testing the ratio against *F*_*n*, *n*_ provides a conservative test of significance. The critical value of *F*_9, 9_(0.99) is 5.3511, indicating that the first two recombinants are significant at the 1% level. In the following, we continue with this example using only these first two (significant) recombinants.

#### Residual plots

Corroboration of the recombinants can be seen by examining ‘residual’ VDV-1 proportion plots. A model with DWV and VDV-1 alone has the form

**Figure 8.**
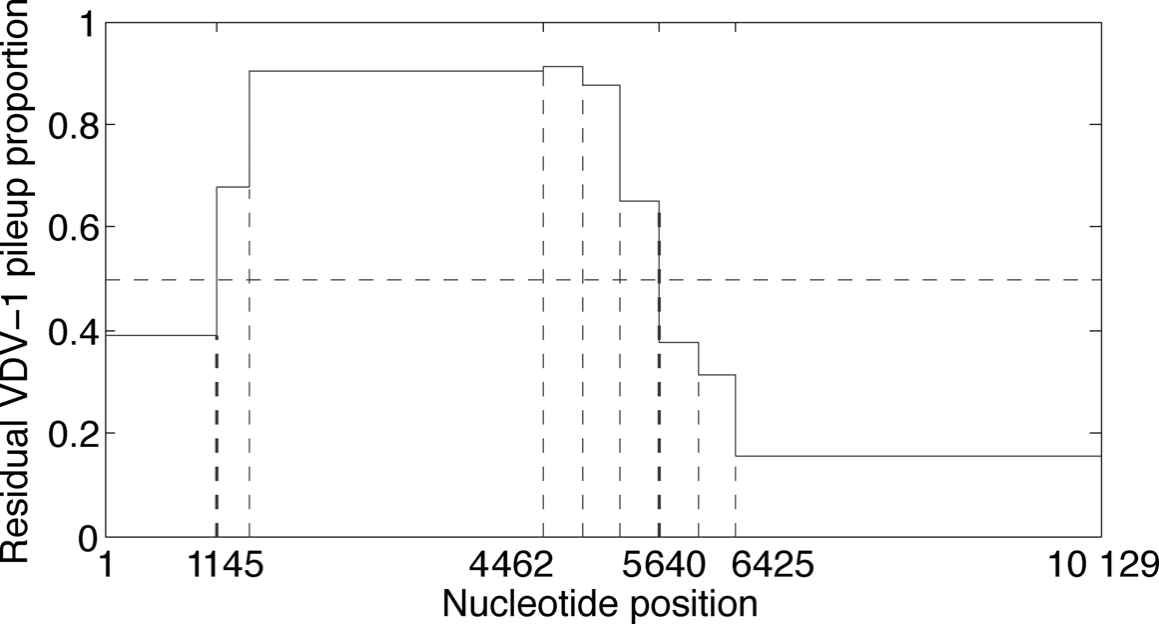
The proportion of VDV-1 unexplained in the data, across segments, after fitting only the DWV and VDV-1 components to the siRNA profile in Figure 6a. The raised proportion of VDV-1 seen from the second to fifth segments justifies the form of M1 seen in Figure 6b.

\begin{equation*} y = 0.4524 (-1, \ldots , -1) + 0.5476(1, \ldots , 1) + r, \end{equation*}

where *r* is the transformed residual corresponding to the residual VDV-1 segment proportions *p* = (1 + *r*)/2 shown in Figure 8. This graphic confirms the choice of M1 in Figure 6b as DWV on the first segment, VDV-1 on the next five segments and DWV again on the final three segments, using 0.5 as a threshold. We remark that the DWV and VDV-1 parental virus proportions reduce significantly to 0.0387 and 0.1142, respectively, when the seven recombinants of Figure 6 are included in the model.

#### Refinement of significant recombinants

**Figure 9.**
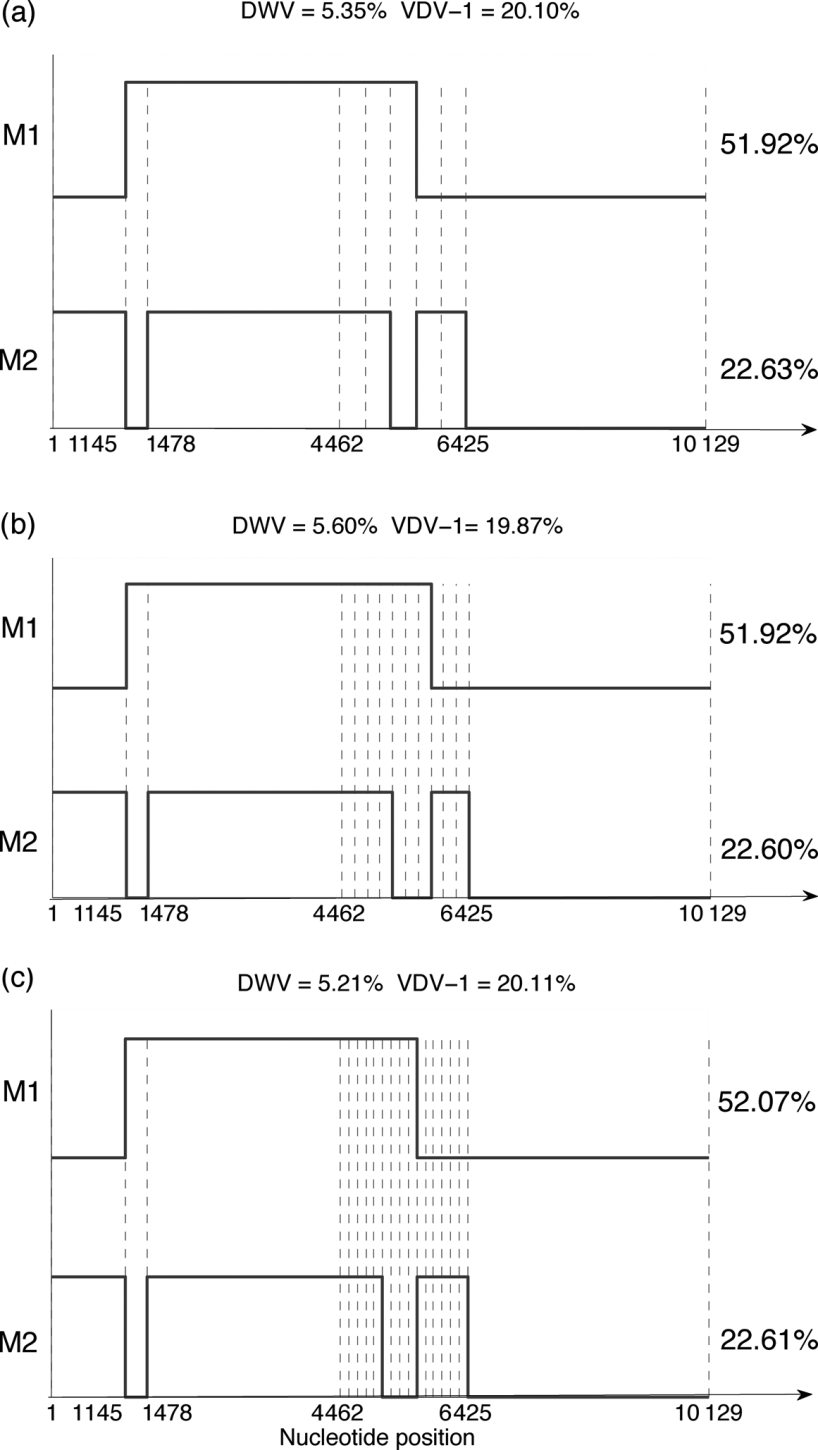
Progressive refinement of the two significant recombinants found in the siRNA data from honeybee pupae. The helicase region is broken into (**a**) five segments, as in Figure 6, (**b**) 10 segments and (**c**) 15 segments. The percentages of DWV and VDV-1 are shown in each case, together with the percentages of each recombinant.

The location of breakpoints in significant recombinants can be refined by progressively adding more breakpoints in regions where recombination is expected or by subdividing existing segments. Figure 9 illustrates such a refinement process, with the helicase region progressively broken into 5, 10 and then 15 segments. Both recombinants M1 are stable. Proportions of DWV, VDV-1, M1 and M2 are stable.

*Example 2: Viral recombinant identification using whole genome short read data (the viral genome data set) with experimental verification of mosaic compositions.* In (3) high-throughput Illumina sequencing and alignment of reads to DWV and VDV-1 reference genomes was used to identify DWV/VDV-1 in viral RNA pooled from 40 capped pupae from a *Varroa*-infested honeybee colony. Analysis of pileup data and sequenced clones suggested that recombination had occurred near Lp and in the helicase. The depth of read coverage fell to zero, or very close, in the 3′ region of VDV-1, from which it was concluded that VDV-1 was present, if at all, in very small quantities. Sequencing of cloned viruses demonstrated the existence of DWV in the viral population together with at least two recombinants. Amplified cDNA fragments, using primers spanning the presumed recombination junction and specific for one or other of the parental genomes, were used to confirm the presence of these recombinants and detected them in six of 11 fragments (partial and full genomes); the first recombinant (named VDV-1_DVD_, indicating components of DWV, VDV-1 then DWV, from 5′ to 3′ end) had recombination points at nucleotide 946 and in the region 5787-5821. The second recombinant (named VDV-1_VVD_, indicating components in order VDV-1, VDV-1 then DWV from 5′ to 3′) had a recombination breakpoint in the helicase region, between nucleotides 5122 and 5153. We stress that the recombination points were determined in biologically cloned genomes.

**Figure 10.**
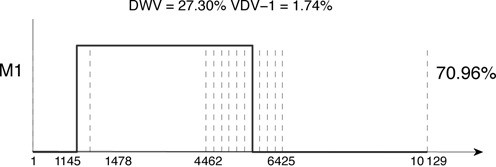
The single significant recombinant (1% level) found in the viral genome sequence data (Illumina) from (3). This recombinant, representing 70.96% of the viral mix, is consistent with that found experimentally. The mix also contained 27.30% DWV and 1.74% VDV-1, largely in agreement with experiment.

We applied our method, using the *n* = 14 segments of Figure 9b, to the ∼2 × 10^7^ cDNA mate-paired reads that aligned uniquely to exactly one of the DWV or VDV-1 genomes. A single significant recombinant at a 1% significance level was found, with DWV comprising 27.30% of the mix and VDV-1 1.74% (Figure 10). This recombinant, with breakpoints at nucleotides 1145 and 5640, accounted for 70.96% of the population, consistent with the VDV-1_DVD_ mosaic detected in the cloned genome, to the accuracy available. Large peaks in the VDV-1 pileup data at the 4600-nt and 5600-nt positions are caught by a second recombinant, although this is not significant, even at the 5% level; thus sequencing depth is insufficient for us to determine at the 5% level of significance the presence of the second mosaic.

*Example 3: Recombinant detection efficacy analysis using simulated data.* Here we simulate viral mixtures comprising mosaics of two parent genomes, with known proportions, and test the accuracy of both the inferred recombinants and their proportions. We generated *m* recombinant mosaics from two parent genomes, with associated weights α = (α_1_, …, α_*m* + 2_). From these weighted recombinants, segment proportions were computed, multiplied by 1000, then rounded to the nearest integer to create pileup counts. An additional normally distributed noise could be added to these counts, the noise having mean zero and standard deviation σ, subject to constraining the resulting counts to be integers in the interval [0, 1000]. Site residuals for the viral pileup experimental data were found to be best described by a *t*_2_ distribution (not shown). The noise added in the simulations is mimicking site residuals summed over genome segments; sums of *t*_2_ values are normally distributed (32), thus justifying the use of normal errors. MosaicSolver was then used to identify the mosaics and their weights. Four examples are presented.

Example 3.1: Single recombinant, no noise added, n = 10, m = 1.

The genomes in the population correspond to the three vertices of *C*^10^ as follows:

**Table:** 

VDV-1	1	1	1	1	1	1	1	1	1	1
DWV	−1	−1	−1	−1	−1	−1	−1	−1	−1	−1
R	−1	1	1	1	−1	−1	−1	1	1	−1

Note that the recombinant *R* contains multiple crossover events. The true proportions α are (0.3271, 0.2865, 0.3864) and the simulated pileup counts are

**Table:** 

VDV-1	287	673	673	673	287	287	287	673	673	287
DWV	713	327	327	327	713	713	713	327	327	713

The recombinant R was found with genome proportions $\hat{\alpha }=(0.3270, 0.2870, 0.3860)$; variation is caused by slight numerical error introduced by the optimization. A single mosaic and associated weights are reliably found for *n* up to the computational limit of the computer used (around *n* = 14 for a MacBook Pro).

Example 3.2: Single recombinant, with noise (σ = 100), n = 10, m = 1.

The viral mix contains three genomes (vertices)

**Table:** 

VDV-1	1	1	1	1	1	1	1	1	1	1
DWV	−1	−1	−1	−1	−1	−1	−1	−1	−1	−1
R	−1	−1	−1	1	1	1	−1	1	1	1

and the true α is (0.3552, 0.5281, 0.1167). Simulated pileup counts are

**Table:** 

VDV-1	504	551	572	583	672	705	537	818	584	571
DWV	548	406	412	373	324	342	532	460	335	388

The recombinant R was still found exactly and $\hat{\alpha }=(0.3768, 0.4907, 0.1326)$. The pattern seen here is general in that the mosaic is found more robustly than are the mosaic proportions. Recombinants are found accurately up to σ = 100, or equivalently up to a coefficient of variation around 20%; beyond that fidelity is progressively lost.

Example 3.3: Three recombinants, serial search, no noise, n = 10, m = 3.

The generated mosaics (DWV and VDV-1 not shown) are

**Table:** 

−1	−1	1	−1	−1	−1	−1	−1	−1	−1
−1	1	−1	1	1	−1	−1	1	−1	−1
1	−1	1	1	1	1	−1	1	−1	1

and the true α is (0.3567, 0.3452, 0.0993, 0.0520, 0.1468). The following mosaics (vertices) were found (note that there is one position in error, shown in bold),

**Table:** 

−1	−1	1	−1	−1	−1	−1	−1	−1	−1
−1	1	**1**	1	1	−1	−1	1	−1	−1
1	−1	1	1	1	1	−1	1	−1	1

and $\hat{\alpha } = (0.4090, 0.3450, 0.0520, 0.0470, 0.1470)$. The transformed segment proportions in *C*^10^ are *y* = (−0.0159, −0.2056, 0.1826, 0.0880, 0.0880, −0.0159, −0.3095, 0.0880, −0.3095, −0.0159) whilst the nearest point found, at a Euclidean distance of 0.001, is *x* = (−0.0160, −0.2060, 0.1820, 0.0880, 0.0880, −0.0160, −0.3100, 0.0880, −0.3100, −0.0160).

Example 3.4: Three recombinants, serial search, with noise (σ = 100), n = 10, m = 3.

The generated mosaics (DWV and VDV-1 again not shown) are

**Table:** 

−1	1	−1	−1	−1	−1	−1	−1	1	1
−1	1	−1	−1	1	1	−1	1	−1	1
1	1	1	1	−1	−1	1	−1	−1	1

and the true α is (0.3430, 0.2603, 0.1792, 0.1435, 0.0741). Simulated pileup counts, including noise, are

**Table:** 

VDV-1	252	648	368	244	375	439	150	508	681	753
DWV	814	257	744	697	573	490	638	587	414	362

Mosaics present at higher levels were detected with higher accuracy (the six incorrect sites are shown in bold) and were

**Table:** 

−1	1	−1	−1	−1	−1	−1	−1	1	1
−1	1	−1	−1	1	1	−1	1	**1**	1
**−1**	1	1	**−1**	−1	**1**	**−1**	**1**	−1	1

with $\hat{\alpha } = ( 0.3010, 0.2334, 0.2271, 0.1549, 0.0837)$. The transformed segment proportions in *C*^10^ are *y* = (−0.3313, 0.3140, −0.3313, −0.3313, −0.1925, −0.1925, −0.3313, −0.1925, −0.1211, 0.3140) whilst the nearest point found, at Euclidean distance 0.5474, is *x* = (−0.5333, 0.3980, −0.3660, −0.5333, −0.2235, −0.0562, −0.5333, −0.0562, 0.2307, 0.3980).

### Blind detection of recombination points

**Figure 11.**
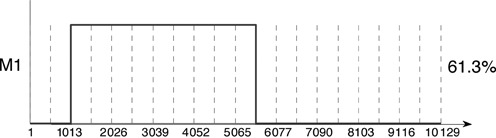
Blind search for recombination points. A single mosaic was found using the siRNA data set and 19 breakpoints creating 20 segments of equal length along the genome. The recombination points could be further refined by application of a finer grid in the neighbourhoods of the located breakpoints, as in Figure 9.

The method can be used for the detection of recombination points when there is no prior knowledge of the location of breakpoints. By way of example, the DWV genome was split into 20 equal segments (each of ∼500 nts) and a single recombinant found using the siRNA pileup data of the Examples on pileup data section. The result is shown in Figure 11. The breakpoints (known to be at 946 nt and ∼5800 nt, as described in Example 2) are found to the accuracy of the subdivision. This profile can also usefully be compared with the first recombinant shown in Figure 9, which used functionally defined blocks (5′ UTR, Lp, capsid, subdivision of the helicase region). The profiles are identical, up to the accuracy of the segmentation.

### Accuracy limits

**Figure 12.**
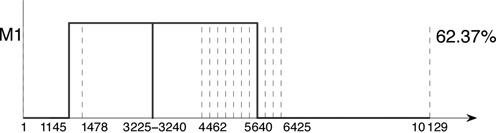
The effect of small segment sizes on the accuracy of the recombinant. When the segment width is reduced to 15 nt in a region chosen, where the VDV-1 pileup proportion falls below 0.5, spurious recombination points are detected.

As the segmentation is refined, noise in the pileup data may give rise to spurious recombination points. In picorna-like viruses, such as DWV, incompatibility of protein–protein interactions in the formation of the viral capsid means that crossover events within the capsid-coding region rarely, if ever, yield viable progeny from recombination between divergent parental genomes (33). For this reason, we use this block to look for the ‘false recombination detection threshold’, again with the siRNA data set. Breakpoints used are as given in Figure 9b together with positions at 3225 nt and 3240 nt within the capsid-coding region, where the proportion of VDV-1 pileups falls briefly below 0.5 to 0.4518. Figure 12 shows the resulting profile when this segment is included; the two spurious recombination points at nt positions 3225 and 3240 are evident. Study of progressively smoothed pileup data (e.g. using a moving average, with increasing window size) and knowledge of segmentation could be used to determine a safe minimum segment width.

This problem does not always occur. In the viral genome data set of Example 2, a larger region, from nt positions 1683 to 1889, has VDV-1 pileup ratio less than 0.5. Despite inclusion of these additional breakpoints in the analysis, the first recombinant stays at VDV-1 throughout the capsid region. A dip of the type shown in Figure 12 only appears in a second recombinant. The reason is that, unlike the siRNA data set of Figure 12, there is essentially no VDV-1 component; hence the first recombinant remains at VDV-1 between nts 1683 and 1889.

### Experimental validation of MosaicSolver predictions

The consistency of our predicted breakpoints with those found in pre-existing cloned fragments was already mentioned in Example 2 (the Examples on pileup data section). Here we discuss additional experimental work that was performed to verify our predictions.

Firstly, analysis of the siRNA data set of Example 1 predicted a recombinant with 5′ region consisting of VDV-1 UTR and CP-coding sequences separated by a DWV Lp coding sequence (Figure 9, recombinant M2). These junctions were at nucleotide positions 1145 and 1478. Using reverse transcriptase-polymerase chain reaction (RT-PCR) with primers (5′-CTGTATGAGGCGAAAGTGTGAAAG-3′ and 5′-CCTTTCGCATGGTCTTTCTTC-3′) we confirmed the presence of such a recombinant by successfully amplifying it from RNA extracts from the honeybee pupae used for the siRNA sequencing. The amplified fragment was cloned and sequenced (GenBank accession number KF164292) and had recombination junctions between nucleotide positions 1183 and 1195 (VDV-1 to DWV at the UTR, Lp junction) and between nucleotide positions 1686 and 1688 (DWV to VDV-1, at the Lp, capsid junction). These are within 38–49 and 208–210 nt, respectively, of the locations found by MosaicSolver. A very similar recombinant viral genome with the same arrangement of VDV-1 and DWV blocks was reported in an independent study (19). The nucleotide sequence alignments of both these recombinants, together with the corresponding regions of DWV and VDV-1, are presented in Supplementary Figure S1.

**Figure 13.**
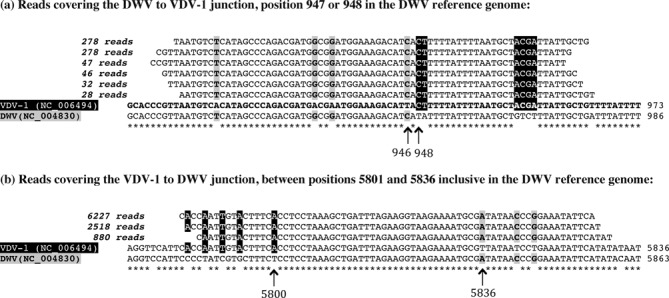
Reads covering recombination junctions (Example 2). (**a**) The DWV to VDV-1 transition. The six main read types are shown, with their frequency listed at the left-hand end. There are sites of variation at DWV positions 946 and 948. Alignments here indicate that up to nucleotide position 946 agreement is with VDV-1, whilst from nucleotide position 948 agreement is with DWV. The recombination junction is therefore at position 947 or 948. (**b**) The VDV-1 to DWV transition. Here straddling sites of variation are further apart, at DWV positions 5800 and 5836. Alignments at these positions indicate that the recombination junction lies between nucleotide positions 5801 and 5836 inclusive. At sites of variation, agreement with VDV-1 is shown in black and agreement with DWV in grey. Sites where DWV and VDV-1 agree are denoted by asterisks.

Secondly, for the viral genome data set of Example 2 (Figure 10) we identified individual sequence reads spanning the predicted recombination junctions, i.e. these reads switch from DWV to VDV-1, or vice versa, along their length. These recombinant reads confirmed that the recombination junctions lie in the intervals 947–948 nt and 5801–5836 nt, respectively (Figure 13). MosaicSolver had found these at nucleotide positions 1145 and 5640, a satisfactory result, given the breakpoints supplied (shown in Figure 10).

Thirdly, in order to check the proportions that were estimated using MosaicSolver for this data set (Example 2), RNA was re-isolated from the viral preparation used in (3) and quantitative PCR (qPCR) used to estimate the proportion of DWV reads in the 5′ UTR, capsid and non-structural regions. These were 87.01%, 26.96% and 100.00%, respectively, compared to our computationally estimated 98.20% (being 27.30% DWV plus 70.90% M1) in the 5′ UTR, 27.30% (DWV alone, since no contribution from M1) in the capsid region and 98.20% (again, from DWV and M1) in the non-structural region. Thus, there is excellent agreement in the capsid and non-structural regions.

Fourthly, in order to assess the accuracy of MosaicSolver proportion predictions we directly analysed NGS results of an experimentally generated 3:1 VDV-1_VVD_ to VDV-1_DVD_ mix (see the Materials and Methods section) of known RNA sequences. MosaicSolver searched the resulting pileup counts for two recombinants, using breakpoints at nucleotides 946, 5138 and 5804, and found 78.2% VDV-1_VVD_, 20.8% VDV-1_DVD_ and 1% VDV-1. Both recombinants were significant at the 1% level. Blind detection of recombination points using 20 equal segments (as in Figure 11) again found VDV-1_VVD_, VDV-1_DVD_ and VDV-1, to the accuracy available, with percentages 74.1%, 23.2% and 2.7%, respectively. Both recombinants were again significant at the 1% level. This is in excellent agreement.

### Identifiability

**Figure 14.**
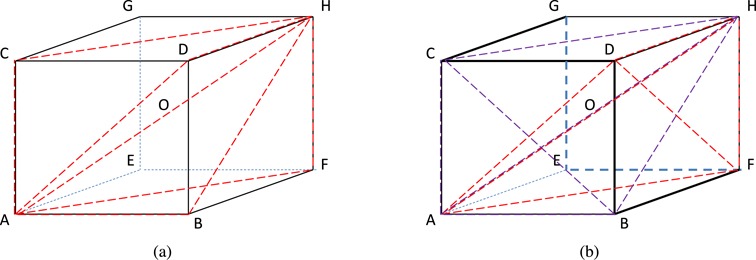
Regions of identifiability and non-identifiability. (**a**) The intersection of all triangles formed by A(=DWV) and H(=VDV-1) and any one of B, C, D, E, F and G is the line AH. The red lines demarcate these triangles for B, C, D and F. Essentially, all points *y* are thus identifiable. (**b**) When two recombinants are sought, points near AH are not identifiable; for example, those points in the intersection of tetrahedra ABCH (purple) and ADFH (red). Points near the darker perimeter edges are identifiable.

When *m* is large, more than one set of *m* recombinants can give rise to a given set of *n* segment proportions. In this case the unravelling problem is not identifiable. Specifically, non-identifiability arises when the *n*-tuple of transformed segment proportions *y* lies in two or more simplexes, each with vertices DWV, VDV-1 and a number of other vertices of the hypercube *C*^*n*^. Convex hulls of more than *n* + 1 vertices of *C*^*n*^ contain overlapping simplexes and hence non-identifiable points. Therefore, a requirement for identifiability is that *m* + 2 ≤ *n* + 1 or *m* < *n*, but this alone does not guarantee identifiability. Figure 14 illustrates this when *n* = 3, where the situation can be visualized. When *m* = 1, illustrated in Figure 14a, the intersection of any two distinct triangles, each determined by DWV (vertex A), VDV-1 (vertex H) and one of the other six vertices, is the diameter AH. Thus for any *y* a single recombinant is identifiable. On the other hand, when *m* = 2, illustrated in Figure 14b, the intersection of a pair of 3-simplexes (each spanned by the DWV, VDV-1 diameter and two vertices other than A and H) can have non-zero measure. Such points, for example, occur near A and H, produced by the intersection of ABCH and ADFH. Points near the CDBFEGC perimeter, on the other hand, are identifiable, lying in a unique 3-simplex.

In general, larger *n* (yielding a more spacious cube) and smaller *m* (yielding simplexes of lower dimension) improve identifiability. For fixed *n* and *m*, identifiability also improves as the data point *y* moves away from the DWV, VDV-1 axis (AH). This confirms the intuition that the more distinct the recombinants are from either DWV or VDV-1, and the more heavily weighted they are, the more readily they can be identified. In the serial algorithm, in order to ensure at each iteration that a unique solution exists, only subsequent vertices for which a solution (when used with vertices already in the model) is unique are considered. For the same reason, for the parallel algorithm only size *m* vertex sets for which the solution is unique are used. A partial resolution of the non-identifiability issue is to record all simplexes whose nearest points to *y* are at the minimum distance found, so providing all possible sets of recombinants (multiple solutions). This has been done for the siRNA data set shown in Figure 6; successive recombinants in this example are found uniquely.

Finally, the way in which identifiability improves as *n* increases and *m* decreases is demonstrated in Table 1. We randomly produced *m* mosaics, each with *n* segments, and their associated proportions, from which exact pileup proportion data were generated. The proportion of correctly found segments (of the *mn* possible) was noted, together with whether the correct mosaic solution was found. Summary results, based on 100 runs, are presented in Table 1, for both serial and parallel search. Single recombinants are found without error whilst individual segments are found with increasing accuracy as *n* increases and *m* decreases.

**Table 1.** A comparison of serial and parallel search

		*m*		
Success rate		1	2	3
	2	1.00	-	-
				
	3	1.00	0.84 (0.30)	-
		1.00	0.84 (0.39)	-
				
	4	1.00	0.90 (0.46)	0.81 (0.02)
		1.00	0.91 (0.63)	0.75 (0.04)
				
	5	1.00	0.91 (0.58)	0.84 (0.05)
		1.00	0.92 (0.59)	0.81 (0.09)
				
*n*	6	1.00	0.93 (0.55)	0.81 (0.09)
		1.00	0.90 (0.58)	0.84 (0.18)
				
	7	1.00	0.93 (0.58)	0.84 (0.11)
		1.00	0.95 (0.80)	0.86 (0.28)
				
	8	1.00	0.93 (0.61)	0.82 (0.14)
		1.00	0.95 (0.79)	-
				
	9	1.00	0.91 (0.61)	0.84 (0.15)
		1.00	0.95 (0.81)	-
				
	10	1.00	0.93 (0.62)	0.83 (0.16)
		1.00	0.98 (0.87)	-

The average proportion of segments found correctly in 100 runs as *n*, the number of segments, and *m*, the number of recombinants (necessarily <*n*), vary. The upper unbracketed number in each cell is for serial ‘stepwise forward’ search and the lower figure for parallel ‘all-at-once’ search. Figures in brackets are the associated proportion of runs in which all recombinants were found exactly. Note that mosaics are more readily found (since identifiability improves) as *n* increases and *m* decreases.

## DISCUSSION

MosaicSolver has a number of dependencies that can affect the solution to the genome composition problem; therefore, a number of issues need to be discussed to obtain the best solution and confidence in that solution. Firstly, there is the choice of breakpoints; these determine the segmentation of the consensus genome and the set of mosaics considered in the search. Breakpoints should ideally be chosen from expert knowledge of recombination hotspots, i.e. located at known recombination points, or between the coding regions of individual proteins, protein domains or functional RNA elements. In genomes that express a single polyprotein such as DWV and other picorna-like viruses, such breakpoints often map to the proteolytic processing junctions that are used to cleave the polyprotein into the individual functional proteins (12). In the absence of expert knowledge, e.g. poorly studied or novel viruses, we recommend the use of an initial uniform segmentation, or one based on the analysis of the pileup data; the method then allows *de novo* detection of regions in the genomes where recombination occurs. In all cases, refinement can then be used to home-in on the breakpoints, dependent on the sampling rate per nucleotide. Ultimately, the mosaic is approximated by recombinants constrained by the applied breakpoints. The method complements direct detection [as in (34)] of recombination breakpoints using individual reads. Secondly, we demonstrated (Table 1) that a parallel search, although computationally expensive, has higher accuracy, and so should be used if possible. The size of the computational search space may however be prohibitive, limiting the user to a serial search. For a MacBook Pro8,1 (2-core, 2.4 GHz, 256 KB cache per core), the serial method comfortably handles up to around 13 breakpoints, whilst the parallel method cannot progress beyond around eight breakpoints. For *m* recombinants and *n* segments, serial search has a complexity linear in *m*, whereas parallel search has an exponential dependence in *m*; i.e. the running time scales as *O*((2^*n*^)*m*) and *O*((2^*n*^)^*m*^), respectively. Potential improvements could be made by reducing the search space, e.g. given *y*, the vertex sets in the half-space of *C*^*n*^ not containing *y* could be eliminated from consideration as the positivity requirement on the genome proportions means they would never be used. Implementing the algorithm in a faster language such as C++ would also considerably improve speed. Thirdly, numerical accuracy of the quadratic program introduces a small error (illustrated in Example 3.3) that may become important when finding a very large number of recombinants. Fourthly, the algorithm is highly flexible as regards data type. Any data providing relative proportions of parental variants at each position (hence any segment) of the genome can be used to infer recombinants, the localization accuracy depending on the sampling rate per nucleotide; for example, NGS data as illustrated here, tiling arrays based on the parental genomes, qPCR cycles or microarray hybridization signal data. A strength of the method is that it overcomes read preference for certain regions (for example, GC-rich), since such heterogeneity along the genome is removed by using (pileup) proportions. The algorithm can also cope with missing or partial data, e.g. in the NGS data if some segment proportions are not available (due to there being no reads in that segment), then the data determine a convex polytope (the intersection of a finite number of half-spaces) in the hypercube. Methods exist to determine the minimum distance between such a generalized data set and the convex polytope of the model space, whence the method can progress in the way that has been described, although this is not implemented in MosaicSolver.

MosaicSolver can be generalized to search for deletion recombinants, such as those present in defective interfering particles (35). These correspond to points (no longer vertices) on the boundary of the hypercube *C*^*n*^ with zero entries in the positions corresponding to the deleted segments and ‘−1’ and ‘1’ entries elsewhere. Non-homologous recombinants, however, would lie in too large a space to search without additional constraints. The recently described biphasic recombination mechanism of human enteroviruses involves the initial generation of imprecise products within defined regions of the virus genome that span the encoded polyprotein proteolytic cleavage sites. We are exploring the application of MosaicSolver to NGS data sets from such recombinants, using knowledge of the clustering of recombination junctions as suitable constraints to reduce the sequence space to be explored.

At present the method is limited to finding recombinants of just two parent genomes, but extension to a larger number of parent genomes is possible. The essence of the method for finding a first recombinant when there are two parent viruses is, firstly, representation of recombinants as the vertices of a hypercube *C*^*n*^, secondly, representation of the segment pileup proportions as a point *y* in the hypercube and, finally, searching of all non-parental vertices for the one which, when combined with the two parent vertices, creates a triangle with a point closest to *y*. In order to generalize this model, we observe that the hypercube is a Cartesian product of intervals (one for each of the *n* consensus viral genome segments), that selection of the same endpoint in each interval represents a parent virus and that a segment pileup proportion can be represented by a point in the associated segment interval. An interval (such as [−1, 1]) is a simplex of dimension one, *S*^1^, with two endpoints. The key idea in generalizing the model to handle *p* > 2 parents is to replace the interval *S*^1^ with a (*p* − 1)-dimensional simplex *S*^*p* − 1^ (so for *p* = 3, a triangle, for *p* = 4, a tetrahedron and so on). All recombinants of *p* parent viral genomes of length *n* are then the vertices of the *n*-fold Cartesian product (*S*^*p* − 1^)^*n*^; this reduces to the hypercube *C*^*n*^ when *p* = 2. Each segment has *p* pileup proportions and these can be represented as a point in the associated component simplex. The vector of these pileup proportions *y* maps the data to the space (*S*^*p* − 1^)^*n*^. A search for the vertex of (*S*^*p* − 1^)^*n*^ that, together with the *p* parent vertices, creates a *p*-dimensional simplex with a point closest to *y* provides the first recombinant. Extension of the method to cover this general situation is planned.

## CONCLUSIONS

We have described a deterministic method that finds viral recombinants of two parent genomes and their respective proportions in a viral mix. The method is flexible as regards both data type and prior information, e.g. the existence of expert data on recombination sites. Accuracy improves as the number of recombination points increases and the number of recombinant genomes in the mix decreases (Table 1), although the number of recombination points is ultimately limited by the sampling rate per nucleotide and the size of the genome because of computational constraints. Two search methods were described, serial and parallel, with serial search being significantly faster but slightly less accurate than parallel search. Serial search in the honeybee context has shown itself to be sufficiently accurate to detect and define biologically relevant recombinants within a mixed virus population. Such predominant virus(es) are likely to be the most important as far as pathogenesis, immune response, replication advantage or transmission are concerned.

We illustrated our methodology on two experimental data sets, inferring the recombination points of DWV, VDV-1 mosaic genomes. We predicted recombinant genomes from siRNA data and viral sequence data; the former reflect the components of a viral complex targeted by RNAi. We found that both data sets had an identical first recombinant, in fact the dominant genome (Figures 9 and 10) with the capsid and part of the helicase VDV-1, and both the 3′ and 5′ ends DWV. This recombinant is the VDV-1_DVD_ recombinant of (3) seen in cloned fragments. The second recombinant, found using the siRNA data, had recombination points near those of the first recombinant, straddling the *Lp* gene and shifted within the helicase (Figure 9); we experimentally validated the Lp-straddling recombination junctions using qPCR. Consistent with previous reports we found that the capsid region appears stable, i.e. recombinants in this region are likely non-viable, whereas the helicase region is a recombinant hotspot. The DWV genome was at low abundance in both data sets, whilst VDV-1 is also present as a minor population. Assuming the viruses have replicated for similar periods this suggests that the predominant recombinant is the fittest strain in the honeybee-mite host system. The parental DWV strain appears out-competed and thus is unlikely to be a determinant in the pathogenic impact of mites on honeybees. The fittest strains have structural components identical to VDV-1, possibly because this expands its tropism or enables the virus to escape the host antiviral response. It is highly probable that DWV evolved to be avirulent utilizing vertical transmission (36) and host/colony survival (37) as a strategy, although this allows more virulent strains, such as a recombinant, a competitive advantage, for instance achieved through a change of transmission dynamics. The virus genome mix, i.e. the relative abundance of the parental and recombinant viral genomes represented in the siRNA data, need not reflect viral genome diversity within the insect hosts; no amplification of the siRNA signal occurs due to the lack of host-encoded RNA-dependent RNA polymerases (38), whilst the RNAi response itself could cause changes in the virus population (39). Despite this, the first recombinant found (Figure 9) is identical to that in the viral sequence data (Figure 10). This indicates that the RNAi response is targeting the dominant viral genotype but appears ineffective at controlling the virus. The recombinant is therefore either highly aggressive or sheltered from the effects of the RNAi response through some unknown mechanism.

In summary, MosaicSolver is a flexible tool for determining the composition and make-up of mixtures of genomes from not only NGS but also any other type of data that profile proportions. Our method can be applied to any mixture of genomes, in particular it can be used to analyse naturally infected organisms, such as humans, where there is inherent host genome heterogeneity; here we applied our method to viral infection in bees from the same colony, so all bees are either full or half-sisters. The method also lends itself to the determination of recombinants of more than two parent viruses, such as those of human, bird and pig, that occur in segmented flu viral reassortments, whilst deletions can in principle be handled by generalizing the methodology. Despite limitations coming from computing power constraining the number of breakpoints to fewer than 15, the method can be applied to as yet poorly characterized (recently discovered novel) viruses, thereby identifying functional blocks that have different selection pressure as regards recombination. By adapting the breakpoints to a region of interest, recombination junctions can also be refined, subject to limits imposed by the experimental data, thereby giving high-resolution detection of recombination junctions.

## SUPPLEMENTARY DATA

Supplementary Data are available at NAR Online.

SUPPLEMENTARY DATA
