# Supplementary material for: MosaicSolver: a tool for determining recombinants of viral genomes from pileup data
Source: Nucleic Acids Res. 2014 Aug 12;42(16):e123. doi: 10.1093/nar/gku524 (PMC4176379; doi:10.1093/nar/gku524)
Supplement: SUPPLEMENTARY DATA [file supp_42_16_e123__index.html]

MosaicSolver: a tool for determining recombinants of viral genomes from pileup data — MosaicSolver: a tool for determining recombinants of viral genomes from pileup data — SUPPLEMENTARY DATA 

# MosaicSolver: a tool for determining recombinants of viral genomes from pileup data

## SUPPLEMENTARY DATA

**Files in this Data Supplement:**

- SUPPLEMENTARY DATA
