# Supplementary material for: MosaicSolver: a tool for determining recombinants of viral genomes from pileup data
Source: Nucleic Acids Res. 2014 Aug 12;42(16):e123. doi: 10.1093/nar/gku524 (PMC4176379; doi:10.1093/nar/gku524)
Supplement: SUPPLEMENTARY DATA [file supp_gku524_FigureS1.pdf]

Figure S1 Nucleotide sequence alignments of the DWV-VDV-1 recombinant genome identified in the UK (KF164292) and the corresponding regions of the DWV (AJ489744), VDV-1 (AY251269) and Israeli DWV-VDV-1 recombinant (JF440526) genomes. Putative regions for the "VDV-1/DWV" recombination junctions (of KF164292 and JF440526) are highlighted in grey and regions for the "DWV/VDV-1" recombination junctions are highlighted in black. The initiation codon for the LP protein and the putative LP-CP cleavage site are shown.

```
DWV-AJ489744         CGAGGCGAAAGTGTGAAAGTTTTGTATGTGTTTTTATATGTACGACTGTATCGGGAATTC 837
VDV-1-AY251269       TGAGGCGAAAGTGTGAAAGTAATTTATGTCTCTATACATAAGTGACTGTATCGGGATTTC 824
RF-KF164292          ------------------GTAATTTATGTCTCTATACATAAGTGACTGTATCGGGATTTC 42
RF-JF440526          TGAGGCGAAAGTGTGAAAGTAATTTATGTCTCTATACATAAGTGACTGTATCGGGATTTC 162
                                       **  * ***** * * ** **    ************* ***
KF164292-dif                             VV V     V V V  V  VVVV             V
JF440526-dif                             VV V     V V V  V  VVVV             V


DWV-AJ489744         CTTTAGCAAGAATCCTTTTAATACAGTATAATTTGTGCTACGGTACGTTACGTTCGCAGG 897
VDV-1-AY251269       CTTTGGCAAGAATCCTTTTAATTCAGTATAATTTATGCTACGGTACGTTACGTTCGCAGG 884
RF-KF164292          CTTTGGCAAGAATCCTTTTAATACAGTATAATTTATGCTACGGTACGTTACGTTCGCAGG 102
RF-JF440526          CTTTGGCAAGAATCCTTTTAATACAGTATAATTTATGCTACGGTACGTTACGTTCGCAGG 222
                     **** ***************** *********** *************************
KF164292-dif             V                 D           V
JF440526-dif             V                 D           V


DWV-AJ489744         GCACCCGTTAATGTCTCATAGCCCAGACGATGGCGGATGGAAAGACATCATATTTTATTT 957
VDV-1-AY251269       GCACCCGTTAATGTCACATAGCCCAGACGATGACGAATGGAAAGACATTACTTTTTATTT 944
RF-KF164292          GCACCCGTTAATGTCACATAGCCCAGACGATGACGAATGGAAAGACATTACTTTTTATTT 162
RF-JF440526          GCACCCGTTAATGTCACATAGCCCAGACGATGACGAATGGAAAGACATTACTTTTTATTT 282
                     *************** **************** ** ************ *  ********
KF164292-dif                        V                V  V            V VV
JF440526-dif                        V                V  V            V VV


DWV-AJ489744         TAATGCTGTCTTTATTGCTGATTTATTTTGCTGTTTTTATTTGCTATTTTATATTTGCTA 1017
VDV-1-AY251269       TAATGCTACGATTATTGCTGTTTTATTTTGCTGTTTTTATTTGTTATTATAT-TTTGCTA 1003
RF-KF164292          TAATGCTACGATTATTGCTGTTTTATTTTGCTGTTTTTATTTGCTATTATAT-TTTGCTA 221
RF-JF440526          TAATGCTACGATTATTGCTGTTTTATTTTGCTGTTTTTATTTGCTATTATAT-TTTGCTA 341
                     *******    ********* ********************** **** *** *******
KF164292-dif                VVVV         V                      D    V
JF440526-dif                VVVV         V                      D    V


DWV-AJ489744         ATTYTCATTATTGCGAAATATATTACATTGCTATTTTTATTATATACGCTAGATTCAATT 1077
VDV-1-AY251269       TT--TTATTATTGCTAAATATATTTCTTTACTATTTTTGCTTTATATATTAGATTCAATT 1061
RF-KF164292          TT-TTCATTATTGCTAAATATATTTCTTTGCTATTTTTGCTTTATATATTAGATTCAATT 280
RF-JF440526          TT-CTCATTATTGCTAAATATATTTCTTTGCTATTTTTGCTTTATATATTAAATTCAATT 400
                      *  * ******** ********* * ** ********  * ****   ** ********
KF164292-dif         V    D        V         V V  D        VV V    VVV
JF440526-dif         V    D        V         V V  D        VV V    VVV


DWV-AJ489744         TTATTTTTYCTATATTTTCAATTTAATTTTGATTTCGAAGGTAAATATATATAATTRATT 1137
VDV-1-AY251269       CTTTTTATTTTATATTTTCAATTTGATTTTGATTTTGAAGGTAAATATATATAA------ 1115
RF-KF164292          CTTTTTATTTTATATTTTCAATTTGATTTTGATTTTGAAGGTAAATATATATAA------ 334
RF-JF440526          CTTTTTATTTTATATTTTCAATTTGATTTTGATTTTGAAGGTAAATATATATAA------ 454
                      * *** *  ************** ********** ******************
KF164292-dif         V V   V  V              V          V
JF440526-dif         V V   V  V              V          V
```

```
                            LP=>
DWV-AJ489744         ATTAAAAATGGCCTTTAGTTGTGGAACTCTYTCTTACTCTGCCGTCGCCCAAGCTCCGTC 1197
VDV-1-AY251269       -----AAATGGCATTTAGTTGTGGAACTCTTTCTTATGCTGCTGTTGCCCAAGCTCCCTC 1170
RF-KF164292          -----AAATGGCATTTAGTTGTGGAACTCTTTCTTATGCTGCTGTTGCCCAAGCTCCGTC 389
RF-JF440526          -----AAATGGCCTTTAGTTGTGGAACTCTTTCTTACTCTGCCGTCGCCCAAGCTCCGTC 509
                          ******* ***************** *****  **** ** *********** **
KF164292-dif                     V                       VV    V  V           D
JF440526-dif                     D                       DD    D  D           D


DWV-AJ489744         TGTCGCYTATGCACCTCGTACATGGGAAGTTGATGAAGCTAGGCGGCGCCGAGTCATTAA 1257
VDV-1-AY251269       TGTAGCTCATGCTCCCCGTAGTTGGGAGATTGATGAAGCTAGGCGTCGACGCGTTATCAA 1230
RF-KF164292          TGTCGCCTATGCACCTCGTACATGGGAAGTTGATGAAGCTAGGCGGCGCCGAGTCATTAA 449
RF-JF440526          TGTCGCCCATGCACCTCGTACATGGGAAGTTGATGAAGCTAGGCGGCGCCGAGTTATTAA 569
                     *** **  **** ** ****  *****  **************** ** ** ** ** **
KF164292-dif            D   D    D  D    DD     DD                D  D  D  D  D
JF440526-dif            D   V    D  D    DD     DD                D  D  D  D  D


DWV-AJ489744         ACGTTTGGCGCTGGAGCAAGAACGTATTCGTAACGTTCTTGACGTTGCCGTCTATGACCA 1317
VDV-1-AY251269       GCGTTTGGCGTTGGAACAGGAACGGATTCGAAACGTTCTTGATGTCACTGTGTATGATCA 1290
RF-KF164292          ACGTTTGGCGCTGGAGCAAGAACGTATTCGTAACGTTCTTGACGTTGATGTCTATGCCCA 509
RF-JF440526          ACGTTTGGCGCTGGAGCAAGAACGTATGCGTAACGTTCTTGACGTTGACGTTTATGACCA 629
                      ********* **** ** ***** ** ** *********** **    ** ****  **
KF164292-ref         D         D    D  D     D     D           D  DDDV        D
JF440526-dif         D         D    D  D     D     D           D  DDDD        D


DWV-AJ489744         GGCGACATGGGAACAGGAGGACGCGCGCGATAATGAGTTCCTAACGGAACAATTAAAYAA 1377
VDV-1-AY251269       TACAACGTGGGAGCAAGAGGATGCGCGTGATAATGAGTTCCTTATGGAACAATTGAATAA 1350
RF-KF164292          GACGACATGGGAACAAGAGGACGCGCGGGATAATGAGTTCCTAACGGAACAATTAAACAA 569
RF-JF440526          GGCGACATGGGAACAGGAGGACGCGCGCGATAATGAGTTCCTAATGGAACAATTAAATAA 689
                       * ** ***** ** ***** ***** ************** * ********* ** **
KF164292-dif         DV D  D     D  V     D                    D D         D
JF440526-dif         DD D  D     D  D     D     D              D V         D


DWV-AJ489744         TTTATATACTATTTATTCGATCGCTGAACGTTGTACGCGTCGGCCTATCAAAGAGYACTC 1437
VDV-1-AY251269       TTTATATACGATATATTCTATAGCTGAAAGATGTACCCGCCGCCCTGTTCAAGAACATGT 1410
RF-KF164292          TTTATATACTATTTATTCGATCGCTGAACGTTGTACGCGTCGGCCCATCAAAGAGCACTC 629
RF-JF440526          TTTATATACTATTTATTCGATCGCTGAACGTTGTACGCGTCGGCCTATCAAAGAGCACTC 749
                     ********* ** ***** ** ****** * ***** ** ** **  *  ****  *
KF164292-dif                  D  D     D  D      D D     D  D  D  DD DD    D  DDD
JF440526-dif                  D  D     D  D      D D     D  D  D  DD DD    D  DDD


DWV-AJ489744         TCCTATATCAGTTTCGAATAGGTTTGCTCCACTGGARTCCCTYAAGGTCGAGGTCGGTCA 1497
VDV-1-AY251269       CCCCATTTCAATCAGTAATAGATATTCCCCTTTAGAATCCCTTAAGATTGAGGTAGGAAA 1470
RF-KF164292          TCCTATATCAGTTTTGAATAGGTTTGCTCCACTGGAATCTCTCAAGGTCGAGGTCGGTCA 689
RF-JF440526          TCCTATATCAGTTTCGAATAGGTTTGCTCCACTGGAATCCCTCGAGGTCGAGGTCGGTCA 809
                      ** ** *** *    ***** * * * **  * ** ** **  ** * ***** **  *
KF164292-dif         D  D  D   D DD D     D D D D  DD D            D D     D  DD
JF440526-dif         D  D  D   D DD D     D D D D  DD D            D D     D  DD
```

```
DWV-AJ489744         AGAAGCARGCGAATGTATRTTTAAGAAACCTAAATATACGCGCRTTTGCAAGAAAGTGAA 1557
VDV-1-AY251269       AGACGCGGGTGAGTTCGTATTTAAGAAACCCAAATATACAAAGATTTGTAAGAAAGTGAA 1530
RF-KF164292          AGAAGCAGGCGAATGTATATTTAAGAAACCTAAATATACGCGCGTTTGCAAGAAAGTGAA 749
RF-JF440526          AGAAGCAGGCGAATGTATATTTAAGAAACCTAAATATACGCGCGTTTGCAAGAAAGTGAA 869
                     *** **  * ** *   * *********** ********     **** ***********
KF164292-dif            D  D  D  D DDD             D        DDDD     D
JF440526-dif            D  D  D  D DDD             D        DDDD     D


DWV-AJ489744         GCGTGTTGCAACTCGCTTCGTTCGTGAAAAAGTTGTTCGTCCTATGTGYTCWAGATCCCC 1617
VDV-1-AY251269       ACGGGTGGCATCAAAATTTGTGCGCGAGAAAGTTGTTAGGCCCGTTTGTAATCGATCGCC 1590
RF-KF164292          GCGCGTTGCAACTCGCTTCGTTCGTGAAAAAGTCGTTCGTCCTATGTGTTCTAGATCCCC 809
RF-JF440526          GCGTGTTGCAACTCGCTTCGTTCGTGAAAAAGTTGTTCGTCCTATGTGTTCTAGATCCCC 929
                      ** ** *** *    ** ** ** ** ***** *** * **  * **     **** **
KF164292-dif         D     D   D DDDD  D  D  D  D         D D  DD D   DD D    D
JF440526-dif         D     D   D DDDD  D  D  D  D         D D  DD D   DD D    D


DWV-AJ489744         TATGCTATTATTTAAGCTTAAGAAAATTATTTATGATTTGCATTTATATAGATTAAGAAA 1677
VDV-1-AY251269       CATGTTATTATTTAAAATTAAGAAAGTAATATATGATCTACATTTGTATCGGTTACGGAA 1650
RF-KF164292          TATGCTCTTATTTAAGCTTAAGAAAATTATTTATGATTTGCACTTATATAGATTAAGAAA 869
RF-JF440526          TATGCTATTATTTAAGCTTAAGAAAGTTATTTATGATTTGCATTTATATAGATTAAGAAA 989
                      *** * ********  ******** * ** ****** * ** ** *** * *** * **
KF164292-dif         D   D          DD        D D  D      D D     D   D D   D D
JF440526-dif         D   D          DD        D D  D      D D     D   D D   D D


DWV-AJ489744         ACAGATTAGGATGTTGAGACGTCAAAAACAGCGCGATTATGAGTTAGAGTGTGTCACTAA 1737
VDV-1-AY251269       ACAAGTTCGGCTTCTCAGACGCGAAAAACAGCGTGAATACGAGTTAGAGTGTGTTACTAG 1710
RF-KF164292          ACAGATTAGGCTTCTCAGACGCGAAAAACAGCGTGAATACGAGTTAGAGTGTGTTACTAG 929
RF-JF440526          ACAGATTAGGTTGTTGAGACGTCAAAAACAGCGCGATTATGAGTTAGAGTGTGTCACTAC 1049
                     ***  ** ** *  * *****  ********** ** ** ************** ****
KF164292-dif            DD  D  V VV V     VV          V  V  V              V    V
JF440526-dif            DD  D    DD D     DD          D  D  D              D    D

                                                                cleavage
                                                            (LP)/(CP)=>
DWV-AJ489744         TCTGYTACAATTATCGAATCCAGTGCAGGCAAAACCAGAGATG GATAACCCTAATCCAGG 1797
VDV-1-AY251269       TTTGCTACAGCTATCTAATCCTGTTTCAGCTAAACCTGAGATG GACAATCCTAATCCTGG 1770
RF-KF164292          TTTGCTACAGCTATCTAATCCTGTTTCAGCTAAACCTGAGATG GACAATCCTAATCCTGG 989
RF-JF440526          TCTGTTACAAATATCGAATCCGGTGCAGGCAAAACCAGAGATG GATAACCCTAATCCAGG 1109
                     * ** ****  **** ***** **    ** ***** ****** ** ** ******** **
KF164292-dif          V       VV    V     V  VVVV  V     V         V  V        V
JF440526-dif          D       DD    D     D  DDDD  D     D         D  D        D


DWV-AJ489744         ACCTGATGGCGAGGGTGAAGTTGAATTAGAAAAGGATAGCAATGTTGTTTTAACAACTCA 1857
VDV-1-AY251269       TCCAGATGGTGAAGGTGAAGTTGAATTAGAAAAGGATAGTAATGTAGTATTAACTACACA 1830
RF-KF164292          TCCAGATGGTGAAGGTGAAGTTGAATTAGAAAAGGATAGTAATGTAGTATTAACTACACA 1049
RF-JF440526          ACCTGATGGCGAGGGTGAAGTTGAATTAGAAAAGGATAGTAATGTTGTTTTAACAACTCA 1169
                      ** ***** ** ************************** ***** ** ***** ** **
KF164292-dif         V  V     V  V                          V     V  V     V  V
JF440526-dif         D  D     D  D                          V     D  D     D  D


DWV-AJ489744         GCGAGATCCTAGTACATCTATTCCAGCGCCGGTGAGCGTAAAATGGAGTAGATGGACTAG 1917
VDV-1-AY251269       ACGTGATCCTAGCACCTCTATTCCTGCTCCAACTAGTGTGAAGTGGAGTAGATGGACTAG 1890
RF-KF164292          ACGTGATCCTAGTACCTCTATTCCTGCTCCAACTAGTGTGAAGTGGAGTAGATGGACTAG 1109
RF-JF440526          GCGAGATCCTAGTACCTCTATTCCTGCTCCAACTAGTGTGAAGTGGAGTAGATGGACTAG 1229
                      ** ******** ** ******** ** **    ** ** ** *****************
KF164292-dif         V  V        D  V        V  V  VVVV  V  V  V
JF440526-dif         D  D        D  V        V  V  VVVV  V  V  V
```

```
DWV-AJ489744         TAATGACGTAGTAGATGATTATGCCACAATCACATCTCGATGGTAYCAGATTGCTGAATT 1977
VDV-1-AY251269       TAATGATGTTGTGGATGATTATGCCACTATAACTTCGCGTTGGTATCAGATTGCCGAATT 1950
RF-KF164292          TAATGATGTTGTGGATGATTATGCCACTATAACTTCGCGTTGGTATCAGATTGCCGAATT 1169
RF-JF440526          TAATGATGTTGTGGATGATTATGCCCCTATAACTTCGCGTTGGTATCAGATTGCCGAATT 1289
                     ****** ** ** ************ * ** ** ** ** ***** ******** *****
KF164292-dif               V  V  V            V V  V  V  V  V              V
JF440526-dif               V  V  V            V V  V  V  V  V              V

DWV-AJ489744         TGTTTGGTCGAAGGATGATCCATTTGATAAGGAGTTAGCACGTTTAATTTTGCCTCGTGC 2037
VDV-1-AY251269       TGTGTGGTCAAAGGATGATCCATTTGATAAGGAATTGGCGCGCTTAATTTTACCTCGAGC 2010
RF-KF164292          TGTATGGTCAAAGGATGATCCATTTGATAAGGAATTGGCGCGTTTAATTTTACCTCGAGC 1229
RF-JF440526          TGTATGGTCAAAGGATGATCCATTTGATAAGGAATTGGCGCGCTTAATTTTACCTCGAGC 1349
                     *** ***** *********************** ** ** ** ******** ***** **
KF164292-dif                  V                       V  V  V  V        V     V
JF440526-dif                  V                       V  V  V  V        V     V

DWV-AJ489744         TTTGTTATCTAGTATAGAGGCTAATTCTGATGCTATATGTGATGTGCCTAATACTATCCC 2097
VDV-1-AY251269       TTTGTTATCTAGTATTGAGGCTAATTCTGACGCTATTTGTGATGTACCTAATACTATTCC 2070
RF-KF164292          TTTGTTATCTAGTATTGAGGCTAATTCTGACGCTATTTGTGATGTACCTAATACTATTCC 1289
RF-JF440526          TTTGTTATCCAGTATTGAGGCTAATTCTGACGCTATTTGTGATGTACCTAATACTATTCC 1409
                     ********* ***** ************** ***** ******** *********** **
KF164292-dif                        V              V     V        V           V
JF440526-dif                        V              V     V        V           V

DWV-AJ489744         ATTTAAGGTACACGCATATTGGCGAGGCGATATGGAAGTTAGAGTTCAAATTAATTCAAA 2157
VDV-1-AY251269       GTTTAAGGTACATGCATATTGGCGTGGAGATATGGAAGTTCGAGTGCAGATTAACTCGAA 2130
RF-KF164292          GTTTAAGGTACATGCATATTGGCGTGGAGATATGGAAGTTCGAGTGCAGATTAACTCGAA 1349
RF-JF440526          GTTTAAGGTACATGCGTATTGGCGTGGAGATATGGAAGTTCGAGTGCAGATTAACTCGAA 1469
                      *********** ** ******** ** ************ **** ** ***** ** **
KF164292-dif         V           V           V  V            V    V  V     V  V
JF440526-dif         V           V           V  V            V    V  V     V  V

DWV-AJ489744         TAAATTCCAAGTTGGTCAATTACAAGCTACTTGGTATTATTCGGATCATGAGAATTTGAA 2217
VDV-1-AY251269       TAAATTCCAGGTTGGTCAATTACAGGCAACTTGGTACTATTCGGATCATGAAAATTTGAA 2190
RF-KF164292          TAAATTCCAGGTTGGTCAATTACAGGCAACTTGGTACTATTCGGATCATGAAAATTTGAA 1409
RF-JF440526          TAAATTCCAGGTTGGTCAATTACAGGCAACTTGGTACTATTCGGATCATGGAAATTTGAA 1529
                     ********* ************** ** ******** *************  ********
KF164292-dif                  V              V  V        V              V
JF440526-dif                  V              V  V        V              V

DWV-AJ489744         TATATCGTCTAAGAGAAGCGTTTATGGATTTTCACAAATGGATCATGCTTTGATTAGTGC 2277
VDV-1-AY251269       TATCCAGACGAAGCGAAGTGTGTATGGTTTTTCGCATATGGATCATGCTTTGATTAGCGC 2250
RF-KF164292          TATCCAGACGAAGCGAAGTGTGTATGGTTTTTCGCATATGGATCATGCTTTGATTAGTGC 1469
RF-JF440526          TATCCAGACGAAGCGAAGTGTGTATGGTTTTTCGCATATGGATCATGCCTTGATTAGCGC 1589
                     ***   * * *** **** ** ***** ***** ** *********** ******** **
KF164292-dif            VVV V V   V    V  V     V     V  V                    D
JF440526-dif            VVV V V   V    V  V     V     V  V                    V

DWV-AJ489744         GTCAGCAAGTAATGAAGCAAAATTAGTTATTCCATATAAGCATGTTTATCCATTTTTACC 2337
VDV-1-AY251269       ATCAGCGAGTAATGAAGCAAAATTAGTGATACCTTTTAAACACGTATATCCATTCTTACC 2310
RF-KF164292          ATCAGCGAGTAATGAAGCAAAATTAGTGATACCTTTTAAACACGTATATCCATTCTTACC 1529
RF-JF440526          ATCAGCGAGTAATGAAGCAAAATTAATGATACCTTTTAAACACGTATATCCATTCTTACC 1649
                      ***** ****************** * ** ** * *** ** ** ******** *****
KF164292-dif         V     V                    V  V  V V   V  V  V        V
JF440526-dif         V     V                    V  V  V V   V  V  V        V
```

```
DWV-AJ489744         GACAAGAATTGTGCCAGATTGGACTACTGGCATTTTAGATATGGGTGCTTTGAACATTCG 2397
VDV-1-AY251269       AACGCGTGTCGTTCCTGATTGGACAACTGGTATTCTTGATATGGGTACCTTAAATATTCG 2370
RF-KF164292          AACGCGTGTCGTTCCTGATTGGACAACTGGTATTCTTGATATGGGTACCTTAAATATTCG 1589
RF-JF440526          AACGCGTGTCGTTCCTGATTGGACAACTGGTATTCTTGATATGGGTACCTTAAATATTCG 1709
                      **  *  * ** ** ******** ***** *** * ********* * ** ** *****
KF164292-dif         V  VV VV V  V  V        V     V   V V         V V  V  V
JF440526-dif         V  VV VV V  V  V        V     V   V V         V V  V  V


DWV-AJ489744         TGTAATTGCTCCCTTACGGATGAGTGCTACTGGTCCAACTACCTGTAATGTCGTCGTGTT 2457
VDV-1-AY251269       TGTAATTGCTCCACTACGTATGAGTGCGACGGGACCAACCACTTGTAATGTTGTAGTATT 2430
RF-KF164292          TGTGATTGCTCCACTACGTATGAGTGCGACGGGACCAACCACTTGTAATGTTGTAGTATT 1649
RF-JF440526          TGTAATTGCTCCACTACGTATGAGTGCGACGGGACCAACCACTTGTAATGTTGTAGTGTT 1769
                     *** ********  **** ******** ** ** ***** ** ******** ** ** **
KF164292-dif                     VV    V        V  V  V     V  V        V  V  V
JF440526-dif                     VV    V        V  V  V     V  V        V  V  D


DWV-AJ489744         TATTAAATTAAATAACAGCGAGTTTACAGGGACTTCTTCTGGTAAGTTTTATGCGAGCCA 2517
VDV-1-AY251269       TATTAAGTTAAATAATAGTGAATTCACTGGCACTTCTTCTGGTAAGTTTTACGCGAATCA 2490
RF-KF164292          TATTAAGTTAAATAATAGTGAATTCACTGGTACTTCTTCTGGTAAGTTTTACGCGAATCA 1709
RF-JF440526          TATTAAGTTAAATAATAGTGAATTCACTGGTACTTCTTCTGGTAAGTTTTACGCGAATCA 1829
                     ****** ******** ** ** ** ** ** ******************** ****  **
KF164292-dif               V        V  V  V  V  V                       V    VV
JF440526-dif               V        V  V  V  V  V                       V    VV


DWV-AJ489744         AATCAGGGCAAAACCTGAGATGGATCGTATATTAAATTTGGCAGAGGGATTGTTGAATAA 2577
VDV-1-AY251269       AATCAGGGCAAAACCTGAAATGGACCGTGTGTTAAATTTGGCAGAAGGATTACTAAATAA 2550
RF-KF164292          AATCAGGGCAAAACCTGAAATGGACCGTGTGTTAAATTTGGCAGAAGGATTACTAAATAA 1769
RF-JF440526          AATCAGGGCAAAACCTGAAATGGACCGTGTGTTAAATTTGGCAGAAGGATTATTAAATAA 1889
                     ****************** ***** *** * ************** *****  * *****
KF164292-dif                           V     V   V V              V     VV V
JF440526-dif                           V     V   V V              V     VV V


DWV-AJ489744         CACGATTGGTGGTAATAATATGGATAATCCTTCTTATCAACAATCTCCTCGTCATTTTGT 2637
VDV-1-AY251269       TACCGTAGGTGGTTGTAATATGGATAATCCGTCATATCAGCAATCTCCGCGTCATTTTGT 2610
RF-KF164292          TACTGTAGGTGGTTGTAATATGGATAACCCGTCATATCAGCAATCTCCGCGTCATTTTGT 1829
RF-JF440526          TACTGTAGGTGGTTGTAACATGGATAATCCGTCATATCAGCAATCTCCGCGCCATTTTGT 1949
                      **  * ******  *** ******** ** ** ***** ******** ** ********
KF164292-dif         V   V V      VV               V  V     V        V
JF440526-dif         V   V V      VV               V  V     V        V


DWV-AJ489744         CCCGACTGGTATGCACAGCTTAGCTTTAGGTACTAATTTAGTTGAACCATTACATGCATT 2697
VDV-1-AY251269       TCCTACTGGTATGCATAGTTTAGCTTTAGGCACTAATTTAGTAGAGCCTTTGCATGCATT 2670
RF-KF164292          CCCTACTGGTATGCATAGTTTAGCTTTAGGCACTAATCTAGTAGAGCCTTTGCATGCATT 1889
RF-JF440526          TCCTACTGGTATGCATAGTTTAGCTTTAGGCACTAATTTAGTAGAGCCTTTGCATGCATT 2009
                      ** *********** ** *********** ****** **** ** ** ** ********
KF164292-dif         D  V           V  V           V           V  V  V  V
JF440526-dif         V  V           V  V           V           V  V  V  V
```

```
DWV-AJ489744         ACGTTTRGATGCAGCCGGTACGACACAACATCCTGTAGGTTGTGCTCCTGATGAAGATAT 2757
VDV-1-AY251269       ACGATTAGATGCATCAGGTACAACACAACATCCAGTTGGGTGTGCGCCTGATGAAGATAT 2730
RF-KF164292          ACGATTAGATGCATCAGGTACAACACAACATCCAGTTGGGTGTGCGCCTGATGAAGATAT 1949
RF-JF440526          ACGATTAGATGCATCAGGTACAACACAACATCCAGTTGGGTGTGCGCCTGATGAAGATAT 2069
                     *** ** ****** * ***** *********** ** ** ***** **************
KF164292-dif            V         V V     V           V  V  V     V
JF440526-dif            V         V V     V           V  V  V     V

DWV-AJ489744         GACTGTATCCTCCATTGCATCTCGATATGGACTAATTAGACGGGTACAATGGAAGAAAGA 2817
VDV-1-AY251269       GACTGTATCTTCCATTGCATCACGATATGGTTTAATTCGCCAAGTGCAATGGAAGAAAGA 2790
RF-KF164292          GACTGTATCTTCCATTGCATCACGATACGGTTTAATTCGCCAAGTGCAATGGAAG----- 2004
RF-JF440526          GACTGTATCTTCCATTGCTTCACGATATGGTTTAATTCGCCAAGTGCAATGGAAGAAAGA 2129
                     ********* ******** ** ***** **  ***** * *  ** *********
KF164292-dif                  V           V        VV     V V VV  V
JF440526-dif                  V           V        VV     V V VV  V
```

B Amino acid sequence alignment of the putative protein encoded by the DWV-VDV-1 recombinant identified in the UK (KF164292) and the corresponding parts of the viral proteins encoded by DWV (AJ489744), VDV-1 (AY251269), and Israeli DWV-VDV-1 recombinants (JF440526)

```
DWV-AJ48974     MAFSCGTLSYSAVAQAPSVAYAPRTWEVDEARRRRVIKRLALEQERIRNVLDVAVYDQAT 60
VDV-1-AY25126   MAFSCGTLSYAAVAQAPSVAHAPRSWEIDEARRRRVIKRLALEQERIRNVLDVTVYDHTT 60
RF-KF164292     MAFSCGTLSYAAVAQAPSVAYAPRTWEVDEARRRRVIKRLALEQERIRNVLDVDVYAQTT 60
RF-JF440526     MAFSCGTLSYSAVAQAPSVAHAPRTWEVDEARRRRVIKRLALEQERMRNVLDVDVYDQAT 60
                ********** ********* *** ** ****************** ****** **   *
KF164292-dif              V         D   D  D                             DV
JF440526-dif              D         V   D  D                             DD


DWV-AJ48974     WEQEDARDNEFLTEQLNNLYTIYSIAERCTRRPIKEXSPISVSNRFAPLESLKVEVGQEA 120
VDV-1-AY25126   WEQEDARDNEFLMEQLNNLYTIYSIAERCTRRPVQEHVPISISNRYSPLESLKIEVGKDA 120
RF-KF164292     WEQEDARDNEFLTEQLNNLYTIYSIAERCTRRPIKEHSPISVLNRFAPLESLKVEVGQEA 120
RF-JF440526     WEQEDARDNEFLMEQLNNLYTIYSIAERCTRRPIKEHSPISVSNRFAPLESLEVEVGQEA 120
                ************ ********************  *  ***  **  *****  ***  *
KF164292-dif                D                    DD  D   D   DD      D   DD
JF440526-dif                V                    DD  D   D   DD      D   DD


DWV-AJ48974     XECXFKKPKYTRXCKKVKRVATRFVREKVVRPMCSRSPMLLFKLKKIIYDLHLYRLRKQI 180
VDV-1-AY25126   GEFVFKKPKYTKICKKVKRVASKFVREKVVRPVCNRSPMLLFKIKKVIYDLHLYRLRKQV 180
RF-KF164292     GECIFKKPKYTRVCKKVKRVATRFVREKVVRPMCSRSPMLLFKLKKIIYDLHLYRLRKQI 180
RF-JF440526     GECIFKKPKYTRVCKKVKRVATRFVREKVVRPMCSRSPMLLFKLKKVIYDLHLYRLRKQI 180
                 *  *******  ********  ********* * ******** ** ************
KF164292-dif      DD       DD        DD         D D        D  D            D
JF440526-dif      DD       DD        DD         D D        D  V            D


                                           <=(LP)  (CP)=>
DWV-AJ48974     RMLRRQKQRDYELECVTNLLQLSNPVQAKPEM  DNPNPGPDGEGEVELEKDSNVVLTTQRD 240
VDV-1-AY251269  RLLRREKQREYELECVTSLLQLSNPVSAKPEM  DNPNPGPDGEGEVELEKDSNVVLTTQRD 240
RF-KF164292     RLLRREKQREYELECVTSLLQLSNPVSAKPEM  DNPNPGPDGEGEVELEKDSNVVLTTQRD 240
RF-JF440526     RLLRRQKQRDYELECVTTLLQISNPVQAKPEM  DNPNPGPDGEGEVELEKDSNVVLTTQRD 240
                * *** *** ******* *** **** *****  ****************************
KF164292-dif     V   V   V       V        V
JF440526-dif     V   V   V                D


DWV-AJ48974     PSTSIPAPVSVKWSRWTSNDVVDDYATITSRWYQIAEFVWSKDDPFDKELARLILPRALL 300
VDV-1-AY251269  PSTSIPAPTSVKWSRWTSNDVVDDYATITSRWYQIAEFVWSKDDPFDKELARLILPRALL 300
RF-KF164292     PSTSIPAPTSVKWSRWTSNDVVDDYATITSRWYQIAEFVWSKDDPFDKELARLILPRALL 300
RF-JF440526     PSTSIPAPTSVKWSRWTSNDVVDDYAPITSRWYQIAEFVWSKDDPFDKELARLILPRALL 300
                ******** ***************** *********************************
KF164292-dif            V
JF440526-dif            V


DWV-AJ48974     SSIEANSDAICDVPNTIPFKVHAYWRGDMEVRVQINSNKFQVGQLQATWYYSDHENLNIS 360
VDV-1-AY251269  SSIEANSDAICDVPNTIPFKVHAYWRGDMEVRVQINSNKFQVGQLQATWYYSDHENLNIQ 360
RF-KF164292     SSIEANSDAICDVPNTIPFKVHAYWRGDMEVRVQINSNKFQVGQLQATWYYSDHENLNIQ 360
RF-JF440526     SSIEANSDAICDVPNTIPFKVHAYWRGDMEVRVQINSNKFQVGQLQATWYYSDHGNLNIQ 360
                ******************************************************  ***
KF164292-dif                                                               V
JF440526-dif                                                               V
```

```
DWV-AJ48974     SKRSVYGFSQMDHALISASASNEAKLVIPYKHVYPFLPTRIVPDWTTGILDMGALNIRVI 420
VDV-1-AY251269  TKRSVYGFSHMDHALISASASNEAKLVIPFKHVYPFLPTRVVPDWTTGILDMGTLNIRVI 420
RF-KF164292     TKRSVYGFSHMDHALISASASNEAKLVIPFKHVYPFLPTRVVPDWTTGILDMGTLNIRVI 420
RF-JF440526     TKRSVYGFSHMDHALISASASNEAKLMIPFKHVYPFLPTRVVPDWTTGILDMGTLNIRVI 420
                 ******** **************** ** ********** ************ ******
KF164292-dif    V        V                V             V            V
JF440526-dif    V        V                V             V            V


DWV-AJ48974     APLRMSATGPTTCNVVVFIKLNNSEFTGTSSGKFYASQIRAKPEMDRILNLAEGLLNNTI 480
VDV-1-AY251269  APLRMSATGPTTCNVVVFIKLNNSEFTGTSSGKFYANQIRAKPEMDRVLNLAEGLLNNTV 480
RF-KF164292     APLRMSATGPTTCNVVVFIKLNNSEFTGTSSGKFYANQIRAKPEMDRVLNLAEGLLNNTV 480
RF-JF440526     APLRMSATGPTTCNVVVFIKLNNSEFTGTSSGKFYANQIRAKPEMDRVLNLAEGLLNNTV 480
                ************************************ ********** ***********
KF164292-dif                                        V          V           V
JF440526-dif                                        V          V           V


DWV-AJ48974     GGNNMDNPSYQQSPRHFVPTGMHSLALGTNLVEPLHALRLDAAGTTQHPVGCAPDEDMTV 540
VDV-1-AY251269  GGCNMDNPSYQQSPRHFVPTGMHSLALGTNLVEPLHALRLDASGTTQHPVGCAPDEDMTV 540
RF-KF164292     GGCNMDNPSYQQSPRHFVPTGMHSLALGTNLVEPLHALRLDASGTTQHPVGCAPDEDMTV 540
RF-JF440526     GGCNMDNPSYQQSPRHFVPTGMHSLALGTNLVEPLHALRLDASGTTQHPVGCAPDEDMTV 540
                ** *************************************** *****************
KF164292-dif      V                                       V
JF440526-dif      V                                       V


DWV-AJ48974     SSIASRYGLIRRVQWKKDHAKGSLLLQLDADPFVEQRIEGTNPISLYWFAPVGVVSSMFM 600
VDV-1-AY251269  SSIASRYGLIRQVQWKKDHAKGSLLLQLDADPFVEQKIEGTNPISLYWFAPVGVVSSMFM 600
RF-KF164292     SSIASRYGLIRQVQWK-------------------------------------------- 556
RF-JF440526     SSIASRYGLIRQVQWKKDHAKGSLLLQLDADPFVEQKIEGTNPISLHWFAPVGVVSSMFM 600
                *********** ****
KF164292-dif               V
JF440526-dif               V
```
